# Unstructured Biology of Proteins from Ubiquitin-Proteasome System: Roles in Cancer and Neurodegenerative Diseases

**DOI:** 10.3390/biom10050796

**Published:** 2020-05-21

**Authors:** Kundlik Gadhave, Prateek Kumar, Shivani K. Kapuganti, Vladimir N. Uversky, Rajanish Giri

**Affiliations:** 1 School of Basic Sciences, Indian Institute of Technology Mandi, VPO Kamand, Himachal Pradesh 175005, India; kundlikgadhave264@gmail.com (K.G.); kumar.prateek3@yahoo.com (P.K.); k.shivanikrishna@gmail.com (S.K.K.); 2 Department of Molecular Medicine and Byrd Alzheimer’s Research Institute, Morsani College of Medicine, University of South Florida, Tampa, FL 33620, USA; vuversky@usf.edu; 3 Institute for Biological Instrumentation of the Russian Academy of Sciences, Federal Research Center “Pushchino Cientific Center for Biological Research of the Russian Academy of Sciences”, Pushchino, 142290 Moscow, Russia

**Keywords:** ubiquitin-proteasome system, intrinsically disordered proteins, protein misfolding, molecular recognition features, cancer, neurodegenerative diseases, protein degradation

## Abstract

The 26S proteasome is a large (~2.5 MDa) protein complex consisting of at least 33 different subunits and many other components, which form the ubiquitin proteasomal system (UPS), an ATP-dependent protein degradation system in the cell. UPS serves as an essential component of the cellular protein surveillance machinery, and its dysfunction leads to cancer, neurodegenerative and immunological disorders. Importantly, the functions and regulations of proteins are governed by the combination of ordered regions, intrinsically disordered protein regions (IDPRs) and molecular recognition features (MoRFs). The structure–function relationships of UPS components have not been identified completely; therefore, in this study, we have carried out the functional intrinsic disorder and MoRF analysis for potential neurodegenerative disease and anti-cancer targets of this pathway. Our report represents the presence of significant intrinsic disorder and disorder-based binding regions in several UPS proteins, such as extraproteasomal polyubiquitin receptors (UBQLN1 and UBQLN2), proteasome-associated polyubiquitin receptors (ADRM1 and PSMD4), deubiquitinating enzymes (DUBs) (ATXN3 and USP14), and ubiquitinating enzymes (E2 (UBE2R2) and E3 (STUB1) enzyme). We believe this study will have implications for the conformation-specific roles of different regions of these proteins. This will lead to a better understanding of the molecular basis of UPS-associated diseases.

## 1. Introduction

Before 1970, lysosomes were thought of as exclusive cellular machinery to execute misfolded protein degradation. However, in 1977, work by Etlinger et al. reported the presence of a second intracellular ATP-dependent mechanism for degradation of proteins [1]. Later, in 1979 and the early 1980s, ATP and the ubiquitin-dependent protein degradation system was discovered by Avram Hershko, Aaron Ciechanover, and Irwin Rose [2,3,4]. This system is currently known as ubiquitin proteasomal system (UPS), and this work earned them the Nobel Prize in Chemistry (2004) [2,3,4]. A protein quality control (PQC) system present in eukaryotic cells is believed to be active in lysosome, UPS, autophagy, and endoplasmic reticulum (ER). In the ER, heat shock proteins (HSPs) bind to misfolded proteins and mediate their refolding. However, misfolded proteins that failed refolding are transported into the cytosol, where they are targeted to UPS or lysosome for their degradation [5,6]. Importantly, UPS plays a vital role in DNA repair, cell growth, immune function, cell-cycle regulation, and numerous non-proteolytic functions, including regulation of histone modification and involvement in vesicular trafficking pathways, and the deregulation in any component of UPS has been associated with several diseases [7,8,9].

**Figure 1:**
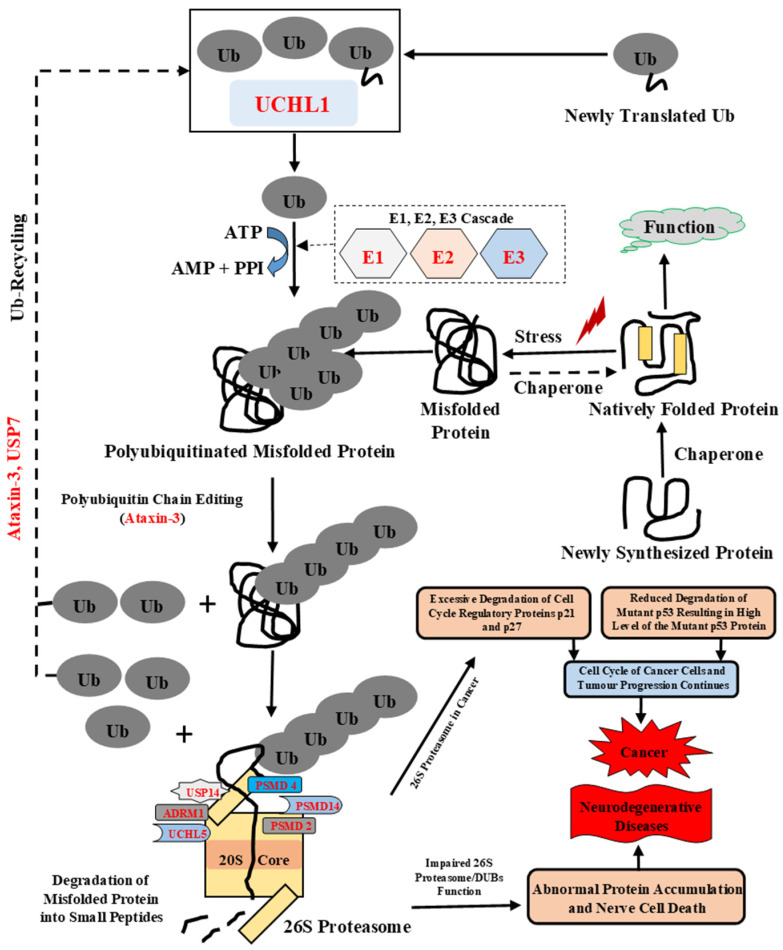
Schematic representation of the ubiquitin proteasomal system. Ubiquitination is an ATP-dependent process performed by three enzymes: E1 (Ub-activating) enzyme, E2 (Ub-conjugating) enzyme, and E3 (Ub-ligase) enzyme. The DUBs, such as ataxin-3, modify the polyubiquitinated chain, to confirm accurate recognition of the misfolded proteins by the 26S proteasome. This covalent modification of misfolded protein targets them to multicatalytic protease complex, the 26S proteasome. Ubiquitination is reversed by DUBs and disassembles polyubiquitin chains. DUBs such as USP7, UCHL1, and ataxin-3 also control and maintain free Ub molecules in the cell. UCHL1 modifies newly translated protein and maintains a pool of mono-Ub. The polyubiquitinated misfolded protein can bind either to the Ub receptor of the 19S regulatory complex or to an adaptor protein that consists of both poly-Ub binding and proteasome binding domain [27]. Once misfolded protein binds to proteasome, the unfolding of the misfolded protein occurs by ATPases followed by removal of the poly-Ub chain by proteasome-associated DUBs and further translocation and degradation of unfolded protein in central proteolytic chamber occurs. Excessive degradation of cell-cycle-regulatory proteins such as p21 and p27 and reduced degradation of mutant p53 leads to a continuous cell cycle of cancer cells and tumor progression leads to the development of cancer [28]. Additionally, impairment in function of 26S proteasome, ubiquitinating enzymes, and DUBs can lead to nerve cell death and the progression of neurodegenerative diseases. Ub: Ubiquitin, E1: Ub-activating enzyme, E2: Ub-conjugating enzyme, E3: Ub-ligase enzyme.

In UPS, the misfolded proteins get ubiquitinated by a cascade of enzymes, including ubiquitin-activating E1s as well as ubiquitin-conjugating E2s and E3 ubiquitin ligases (Figure 1). The ubiquitinated substrates are then transferred to the 26S proteasome for degradation [10,11]. 26S proteasome (~2.5 MDa) is the major protease inside the cell, and has two sub-complexes: the 20S core particle (CP), which is responsible for the main proteolytic activity of the proteasome, and the 19S regulatory particle (RP), which helps in the unfolding of ubiquitinated proteins, their subsequent de-ubiquitination, and their translocation into the CP cavity [12]. The specific lysine (K) residues are required for the conjugating of ubiquitin (Ub) to the substrate proteins, including K6, K11, K27, K29, K33, K48, K63, or Met1 [10,13]. This process results in the generation of either monoubiquitinated, multi-monoubiquitinated, or polyubiquitinated proteins, with these different modes of ubiquitylation determining the fate of the target protein—i.e., degradation of a protein via the 26S proteasome, targeting it to a lysosome or prompting it for participation in other cellular processes [13,14]. Ubiquitin (Ub), a 76-amino-acid ubiquitously distributed polypeptide, is required to tag target proteins for proteasome-mediated degradation [2,15]. Mutations in the *UBB* gene encoding ubiquitin-B and molecular misreading of this gene that introduces dinucleotide deletions (e.g., ΔGA, ΔGU) can generate mutated Ub forms, which are associated with human diseases. For example, the UBB+1 (Ubiquitin-B+1) form of Ub generated as a result of molecular misreading is linked to Alzheimer’s disease (AD), other tauopathies, and polyglutamine (PolyQ) diseases (e.g., Huntington’s disease (HD)) [16,17,18], with the resulting UBB+1 form being shown to inhibit proteasomal proteolysis [19]. These UBB+1 mutants were found with Aβ accumulations in Alzheimer’s and Down syndrome patients [18]. Ub-activating (E1) enzyme catalyzes the first step of ubiquitin activation in the ubiquitination process, where it binds to Ub and transfers it to E2 enzyme [20]. Missense mutations in the *UBA1* gene lead to X-linked spinal muscular atrophy (SMAX2), and reduced UBA1 levels affect UPS-mediated degradation of misfolded proteins leading to neurodegenerative diseases, such as AD [21]. Ubiquitin-conjugating (E2) enzyme catalyzes the second step of ubiquitination, where it accepts Ub from E1 enzyme and transfer it to substrate protein via E3 enzyme [22]. Studies have shown that the impairments of the E2 enzymes or mutations in these proteins are associated with many diseases, such as cancer and neurodegenerative diseases [23]. Ubiquitin-protein ligase (E3) enzyme catalyzes the last step of ubiquitination. E3 binds to a target protein and transfers Ub from the E2 to the target protein. Deregulation of this enzyme is linked to numerous neurodegenerative diseases (AD, Parkinson’s disease (PD), Huntingtons disease (HD) and various cancers [24]. Ubiquilins are functionally linked to UPS, where they act as ubiquitin receptors [25]. The human genome encodes four ubiquilin genes, *UBQLN1*, *UBQLN2*, *UBQLN3*, and *UBQLN4*, which encode structurally related and conserved proteins. A fifth ubiquilin gene, UBQLNL, was later identified in humans. Although all ubiquilin family members are present in cytosol, they exert different tissue expression patterns [25]. Ubiquilin 1 is expressed ubiquitously and binds numerous cytosolic or transmembrane proteins, and its dysfunction is linked to neurodegenerative diseases such as AD, PolyQ diseases (e.g., HD), and cancer [6]. Ubiquilin 2 is associated with the regulation of pathways involved in protein degradation, such as UPS, the endoplasmic-reticulum-associated protein degradation (ERAD) pathway, and autophagy. Interestingly, the mutation in *UBQLN2* was recently described in familial amyotrophic lateral sclerosis (ALS) [26].

Ubiquitination and deubiquitination are dynamic processes that involve transient protein–protein interactions. There are ~100 deubiquitinating enzymes (DUBs) in the human genome that control several cellular processes in a very dynamic and specific manner. Among these DUB-controlled processes are the progression of the cell cycle, degradation of proteins, apoptosis, activation of kinases, chromosome segregation, gene expression, protein localization, and DNA repair [29]. In the UPS, DUBs are involved in several processes, including de-novo ubiquitin synthesis; ubiquitin precursor processing; cleavage and trimming of polyubiquitin chains; and ubiquitin recycling [30]. Ubiquitin carboxyl-terminal hydrolase isozyme L1 (UCHL1) is a small 223-amino-acid protein, which maintains the pool of mono-Ub required for ubiquitination and is also involved both in the processing of ubiquitin precursors and ubiquitinated protein [31]. The mutation I93M in UCHL1 was reported in PD patients. Furthermore, studies in animal models showed that this mutation led to the inhibition of α-synuclein degradation via the 26S proteasome [32]. Ubiquitin carboxyl-terminal hydrolase isozyme L5 (UCHL5) is a crucial DUB enzyme associated with the 19S regulatory subunit of the 26S proteasome that cleaves the Poly-Ub chain of the target protein [33].

The ubiquitin-specific protease (USP) family of DUBs, with more than 50 members, is the largest family among all DUBs [29]. Ubiquitin carboxyl-terminal hydrolase 7 (USP7) is a 135-kDa DUB enzyme that cleaves ubiquitin from the polyubiquitin chains of target proteins [10,34]. It is associated with many cellular processes, and its dysfunction leads to various pathological conditions, such as cancer, metabolic, and neurological pathologies [10]. Ubiquitin carboxyl-terminal hydrolase 14 (USP14) is a proteasome-associated DUB enzyme, which is activated after the specific association with 26S proteasome and catalyzes the cleavage of ubiquitin subunits from the target protein before its degradation by the proteasome [35,36]. Interestingly, USP14 reduces the degradation of pathogenic/toxic proteins (tau protein, TDP-43, α-synuclein) by the 26S proteasome and is therefore associated with many neurodegenerative diseases, such as AD, ALS, PD, and HD, etc. [36]. 26S proteasome non-ATPase regulatory subunit 14 (PSMD14) is a 310-residue DUB enzyme important for Ub recycling from the proteasome substrate proteins, and its upregulation has been reported in cancer, where it promotes proliferation and migration of cancer cells [37,38]. PolyQ expansion in the C-terminus of ataxin-3 results in conformational changes that further lead to altered subcellular localization, loss of function and binding properties, changed proteolytic cleavage, and aggregation of ataxin-3 [39]. Three receptors of the 26S proteasome in the RP, including Proteasomal ubiquitin receptor ADRM1 (ADRM1), 26S proteasome non-ATPase regulatory subunit 2 (PSMD2), and 26S proteasome non-ATPase regulatory subunit 4 (PSMD4) capture the target protein by binding to Ub and target protein shuttle factors [40,41]. The poly-Ub chains of the target protein are cleaved by the 19S-associated DUBs enzymes (PSMD14, USP14 and UCHL5), and then target protein is unfolded and translocated into the CP for its degradation [41,42]. The pathological conditions associated with the UPS occur due to either gain of function leading to abnormal or enhanced degradation of the target protein or due to loss of function mutations in the enzymes of UPS or in the recognition motif in the target substrates that stabilizes certain proteins [43].

Intrinsically disordered proteins (IDPs) and IDP regions (IDPRs) are the proteins or regions of proteins that lack well-defined, three-dimensional unique structures and show structural transition upon binding with their biological partners [44,45,46,47,48,49]. IDPs/IDPRs are commonly found in all organisms, being more abundant in eukaryote proteomes [50,51,52]. Studies have shown that IDPs play a crucial role in protein–protein interaction [53,54,55]. Our previous study on intrinsic disorder analysis of amyloid cascade signaling of AD reports the presence of abundant intrinsic disorder in most of the proteins [56]. Using bioinformatics analysis, previous studies have reported the presence of intrinsic disorder in ubiquitinating enzymes [57,58,59]. This paper covers extensive intrinsic disorder and MoRF analysis of 15 proteins, which are decisive for the functioning of UPS, uniquely associated with the clearance of target proteins, and which are important therapeutic targets for cancer as well as neurodegenerative diseases. Numerous mutations in genes that encode proteins involved in the UPS are linked to such diseases. Proteasome inhibitors, such as bortezomib, carfilzomib, and ixazomib, have been approved in 2003, 2012, and 2015, respectively, for the treatment of certain hematological cancers, and some inhibitors are in clinical trials [60]. Several neurodegeneration-related proteins, such as Aβ, Tau, and α-synuclein, are intrinsically disordered, and distorted protein–protein interactions of those proteins are the main process in the disease pathology [61]. Deciphering the roles of intrinsic disorder in UPS proteins in cellular processes, such as protein–protein interaction, protein recognition, protein degradation, and various signaling pathways could provide important knowledge needed for further advances in the development of new drug targets for cancer and neurodegenerative diseases. Therefore, this work may help for the future establishment of new therapeutic routes for cancer and protein misfolding diseases.

## 2. Materials and Methods

### 2.1. Retrieval of Sequences and Structures

The 15 proteins of UPS that play a crucial role in this pathway and have been identified in the pathogenesis of human diseases were selected for disorder analysis, which is summarized in Table 1. For sequence-based disorder analysis, the reviewed protein sequences of all proteins have been retrieved in the FASTA format from the UniProt [62] database (Table 1), and their associated crystal structures were fetched from the RCSB PDB database. The crystal structures of some proteins are available in truncated form. The IDPs and MoRF regions in the available structures have been represented in different colors.

### 2.2. Identification of Intrinsically Disordered Protein Regions (IDPRs)

Several commonly used disorder predictors, such as PONDR^®^ VSL2 [77], PONDR^®^ VL3 [78], PONDR^®^ VLXT [79], PONDR^®^ FIT [80], and IUPred [81] were utilized for the intrinsic disorder analysis. The mean PPID was calculated for each protein by considering the outputs of five predictors. These predictors use artificial neural networks (ANN) and machine-learning-based algorithms to predict specific disorder regions. The detailed description of the functioning of these predictors has been explained in our previous studies [82,83,84,85,86].

### 2.3. Molecular Recognition Features (MoRFs) Prediction

The MoRFs are disorder binding motifs that are found in specific disordered regions. These regions were predicted using four different web servers, MoRFCHiBi_Web [87], ANCHOR [88], MoRFpred [89], and DISOPRED3 [90]. Each predictor uses a different data sets and ANN-based models for prediction, which are described in our MoRF-based studies on Zika virus, Chikungunya virus, Rotavirus, SARS-CoV-2 proteomes, and Alzheimer’s-disease-associated amyloid cascade signaling proteins [56,83,84,91,92]. Along with these, another web-based predictor, D2P2 [93] has also been used, which predicts disordered regions as well as motifs in proteins.

### 2.4. Protein–Protein Interaction Using STRING

The functioning of disordered regions from important proteins of UPS are explained in the current study. Furthermore, to explore the interaction among these proteins and with other proteins, they have been analyzed using the STRING v11 database [94]. The database contains experimentally determined information on protein–protein interaction as well as computationally predicted possible interactions, and these are constructed as a map.

### 2.5. Representation of IDPs and MoRFs

The predicted IDP regions based on mean PPID value and MoRFCHiBi_Web-predicted MoRF regions were shown on available crystal or NMR structures using the Maestro GUI (v11, Schrödinger Inc., Menlo Park, NY, USA). All the color schemes for representing these regions are given in respective figure legends.

## 3. Results and Discussion

### 3.1. Intrinsic Disorder in the Proteins of Ubiquitin Proteasomal System

UPS is employed by the eukaryotic cell to get rid of excess, unnecessary, or misfolded short-lived regulatory proteins [95]. Figure 1 represents the complete process of ubiquitination and proteasome-mediated degradation of the proteins. The 26S proteasome complex is assembled symmetrically with the 20S CP at the center flanked by two 19S RPs on either side [12]. The 20S particle has a cylindrical structure and is formed by four stacked heptameric rings comprised of the following subunits: α_1–7,_ β_1–7,_ β_1–7_ and α_1–7_. This is 28 subunits in total. β_1_ has caspase-like, β_2_ has trypsin-like, and β_5_ has chymotrypsin-like peptidase activities [96]. These sites are hidden inside the CP to protect cellular proteins from nonspecific degradation. N-termini of the α subunits form the gates to the cylinder. Six ATPases are present in the RP (700 kDa). RP has 19 subunits that are divided into the base and lid complexes. The RPN11/PSMD14 subunit of the lid has deubiquitinating activity, whereas the AAA-ATPases form a hexameric (trimer of dimers) ring in the base. This ring is formed by RPT1–6 proteins. Apart from these, RPN1/PSMD2, RPN2, RPN10/ PSMD4, and RPN13/ADRM1 also form the base complex. RPN10/PSMD4 and RPN13/ADRM1 act as receptors for ubiquitin, whereas RPN1/PSMD2 and RPN2 form a toroid structure and act as a scaffold by binding to different subunits of the proteasome [97,98,99].

Here, we looked at the intrinsic disorder predisposition of 15 UPS proteins (Table 2), which play a crucial role in this pathway and human diseases (see Table 1). These proteins include Ub, ubiquitinating enzymes (E1, E2, and E3), DUBs (USP7, Ataxin-3, UCHL5, UCHL1, USP14, and PSMD14), a receptor for Ub in 26S proteasome (ADRM1, PSMD2, and PSMD4), and ubiquilins (UBQLN1 and UBQLN2), which are involved in the pathogenesis of diseases and are listed in Table 1 with their role in the normal functioning of UPS as well as altered function of an individual protein in UPS and further effects.

**Figure 2:**
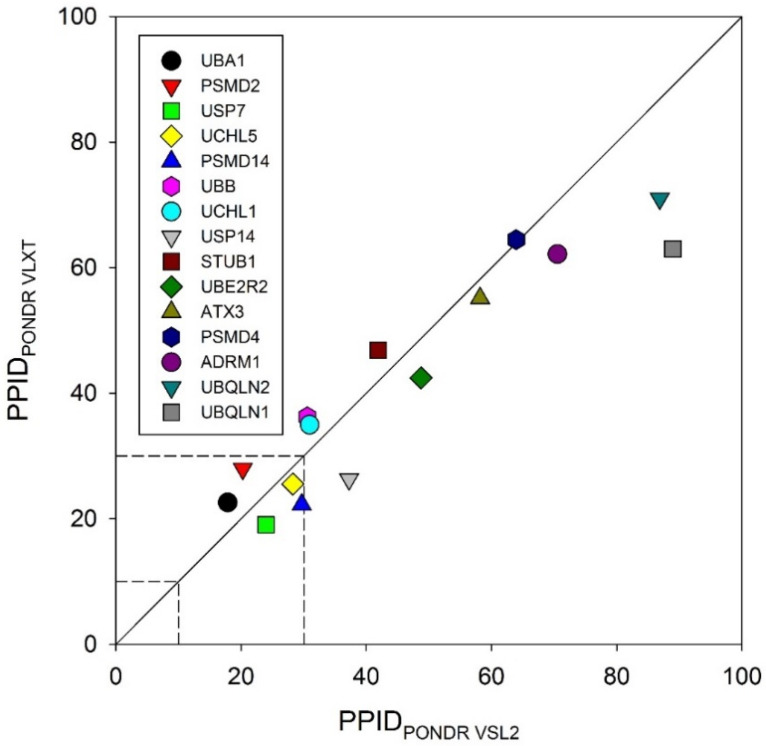
Evaluation of the overall disorder status of 15 UPS proteins associated with human diseases. Here, a 2D disorder plot presents the PPID_PONDR VLXT_ vs. PPID_PONDR VSL2_ dependence.

To obtain a global overview, we looked at the predicted percentage of intrinsic disorder (PPIDs) in these proteins evaluated by PONDR^®^ VLXT and PONDR^®^ VSL2. The results of these analyses are summarized in Figure 2 in the form of the 2D-disorder plot presenting the PPID_PONDR VLXT_ vs. PPID_PONDR VSL2_ plot. According to the overall levels of intrinsic disorder, proteins can be classified as highly ordered (PPID < 10%), moderately disordered (10% ≤ PPID < 30%) and highly disordered (PPID ≥ 30%) [100]. From this broadly accepted PPID-based classification of proteins and mean PPID obtained from five different IDP prediction tools, UBA1 and UCHL1 are highly ordered; USP14, PSMD14, UCHL5, PSMD2, USP7, and UBB are moderately disordered; and UBQLN1, UBQLN2, ADRM1, PSMD4, ATXN3, STUB1, and UBE2R2 are highly disordered proteins. However, from the 2D disorder plot shown in Figure 2, it is clear that only a few UPS proteins analyzed in this study are moderately disordered proteins, with the remaining members of this set being highly disordered. These results indicate the possible role of intrinsic disorder in the pathogenesis of various proteasome-associated diseases.

We also utilized D^2^P^2^ database (http://d2p2.pro/) [93] to retrieve additional information on the intrinsic disorder predisposition together with important disorder-related functional information for the members of the set of 15 UPS proteins associated with human diseases. D^2^P^2^ is a database of predicted disorders for a large library of proteins from completely sequenced genomes. D^2^P^2^ uses outputs of IUPred [81], PONDR^®^ VLXT [79], PrDOS [101], PONDR^®^ VSL2 [77], PV2 [93], and ESpritz [102]. The output of this database is further enhanced by data related to the location of various posttranslational modifications (PTMs) and predicted disorder-based protein binding sites, known as MoRFs. The D^2^P^2^-generated functional disorder profiles of 15 UPS proteins are discussed below for individual proteins. Overall, this shows that all these proteins contain noticeable levels of intrinsic disorder, are heavily decorated with various PTMs, and many of them contain multiple MoRFs, suggesting that these proteins are expected to be characterized by high binding promiscuity. Additionally, the disorder-based protein-binding regions/MoRFs for individual proteins, identified by MoRFCHiBi_Web, MoRFpred, DISOPRED3, and ANCHOR, are listed in Table 3. The MoRFs identified by MoRFCHiBi_Web are represented in the available crystal structure of all proteins (see figures of individual proteins).

**Figure 3:**
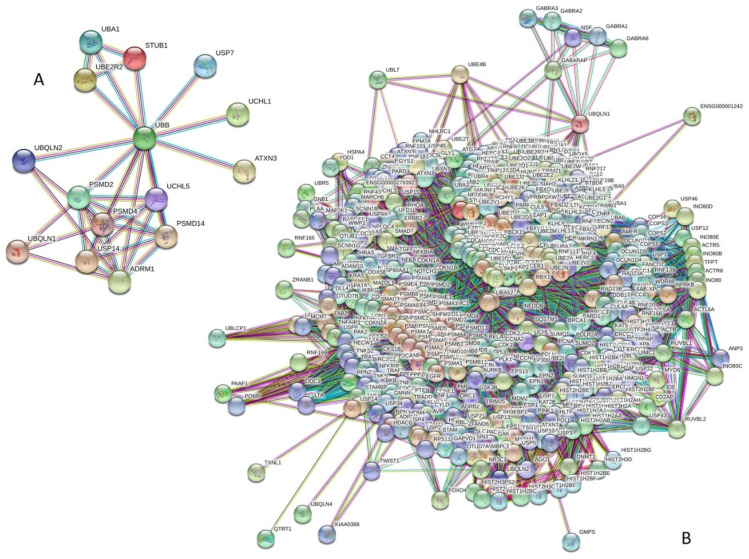
Evaluation of the intractability of 15 UPS proteins associated with human diseases by STRING platform. (**A**) Network of the inter-set PPI interactions (15 subunits, highest confidence level of 0.9). (**B**) PPI network centered at 15 UPS proteins associated with human diseases (515 proteins, highest confidence level of 0.9).

Next, we analyzed the inter-set intractability of 15 UPS proteins associated with human diseases using a publicly available computational platform, STRING, which integrates extensive information on protein–protein interactions (PPIs), complements it with computational predictions, and returns a PPI network showing all possible PPIs of a query protein(s) [94]. The results of this analysis are represented in Figure 3A, which shows that these proteins are engaged in the formation of a highly interconnected PPI network with 36 edges. In this network, the average node degree is 4.8, and the average local clustering coefficient (which defines how close its neighbors are to being a complete clique; the local clustering coefficient is equal to 1 if every neighbor connected to a given node *N_i_* is also connected to every other node within the neighborhood, and is equal to 0 if no node that is connected to a given node *N_i_* connects to any other node that is connected to *N_i_*) is 0.895. Furthermore, since the expected number of interactions among proteins in a similar size set of proteins randomly selected from human proteome is equal to 5, the inter-set PPI network has significantly more interactions than expected, being characterized by a PPI enrichment *p*-value of <10^−16^.

We also used STRING to study the engagement of the subunits of 15 UPS proteins in interactions with 500 proteins forming the first shell of the resulting interactome (note that the number of interactors in STRING is limited to 500). In this analysis, the highest confidence level of 0.9 was used. Figure 3B represents the resulting interactome, which includes 515 nodes connected by 21,801 edges. Therefore, this interactome is characterized by an average node degree of 84.7 and shows an average local clustering coefficient of 0.782. The expected number of interactions for the set of human proteins of this size is 7780, indicating that this PPI network, centered at 15 UPS proteins associated with human diseases, has significantly more interactions than expected (PPI enrichment *p*-value is <10^−16^).

Most of the proteins associated with diseases, such as cancer, AD, PD, diabetes, and cardiovascular disease are either IDPs or contain long IDPRs, and misbehavior or mutations in the IDPs/IDPRs have broad involvement in the pathogenesis of these diseases [44,45,46,47,48,49]. From overall analysis, we found that most of the UPS proteins are intrinsically disordered and are closely linked with the pathophysiology of many diseases, such as cancer, AD, PD, ALS, HD, etc. (see Table 1, Table 2 and Table 3). The proteasomal ubiquitin receptors, such as ADRM1 and PSMD4, and extraproteasomal ubiquitin receptors, such as UBQLN1 and UBQLN12, have been found to be highly disordered as compared with the ubiquitinating and DUB enzymes. These receptors contain several disorder-based protein binding regions, which are involved in protein–protein interaction/molecular recognition. Additionally, several PTMs are located within the disordered regions of these receptors. Furthermore, ubiquitinating enzymes, such as UBE2R2 (E2) and STUB1 (E3), considered important drug targets for cancer and neurogenerative diseases, were also found to be highly disordered, along with many MoRFs and PTMs in disordered regions. Therefore, this study identified highly disordered proteins or proteins with functional DPRs from UPS, which may have a crucial role in the progression of diseases.

#### 3.1.1. Intrinsic Disorder in Ubiquitin-Activating Enzyme (E1 Enzyme)

**Figure 4:**
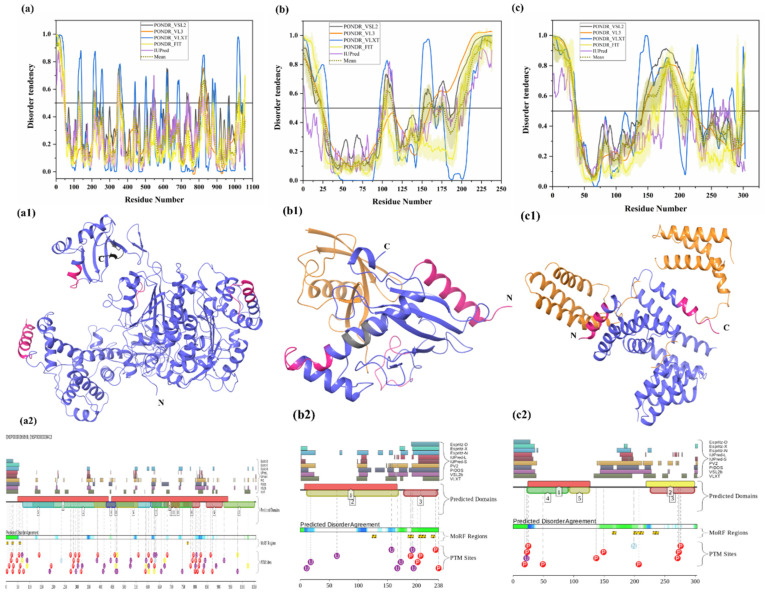
Intrinsic disorder in ubiquitinating enzymes E1 (UBA1), E2 (UBE2R2) and E3 (STUB1). (**a**) ubiquitin-activating enzyme E1 (UniProt ID: P22314), (**a1**) crystal structure of the E1 enzyme (PDB ID: 6DC6). (**b**) ubiquitin-conjugating enzyme E2 (UniProt ID: Q712K3), (**b1**) crystal structure of E2 enzyme having residues 1–202 (PDB ID: 6NYO). (**c**) E3 ubiquitin ligase (UniProt ID: Q9UNE7), (**c1**) crystal structure of E3 ubiquitin ligase (PDB ID: 4KBQ). In Plots (**a**–**c**), the outputs of PONDR^®^ VSL2, PONDR^®^ VL3, PONDR^®^ VLXT, PONDR^®^ FIT, and IUPred are represented by black, orange, blue, yellow, and purple lines, respectively. Mean disorder profile, calculated by averaging the outputs of five predictor-specific per-residue disorder profiles, is depicted by olive color. Light-olive shadow around the mean curve represents the error distribution for the mean. The light-yellow shadow around the PONDR^®^ FIT curve shows error distribution for PONDR^®^ FIT. In (**a1**), crystal structure of the E1 enzyme (PDB ID: 6DC6) is represented in faded blue color, disordered residues are shown in salmon pink color, and MoRF residues identified by MoRFCHiBi_Web server (1048–1057) are shown in grey color. In (**b1**), E2 enzyme (1–202 length with missing residues 1–5 and 193–202 at N- and C-terminal, respectively) is shown with Ubiquitin-60S ribosomal protein L40 (RPL40A; orange color). In (**c1**), Hsc70 Lid-Tail domains (orange color) in complex with E3 ubiquitin ligase (which is represented in faded blue color); disordered residues in E3 are shown in salmon pink color. In (**a2**,**b2**,**c2**), functional disorder profiles, MoRFs, and PTMs in E1, E2, and E3 enzymes using D2P2 server have been shown.

**Figure 5:**
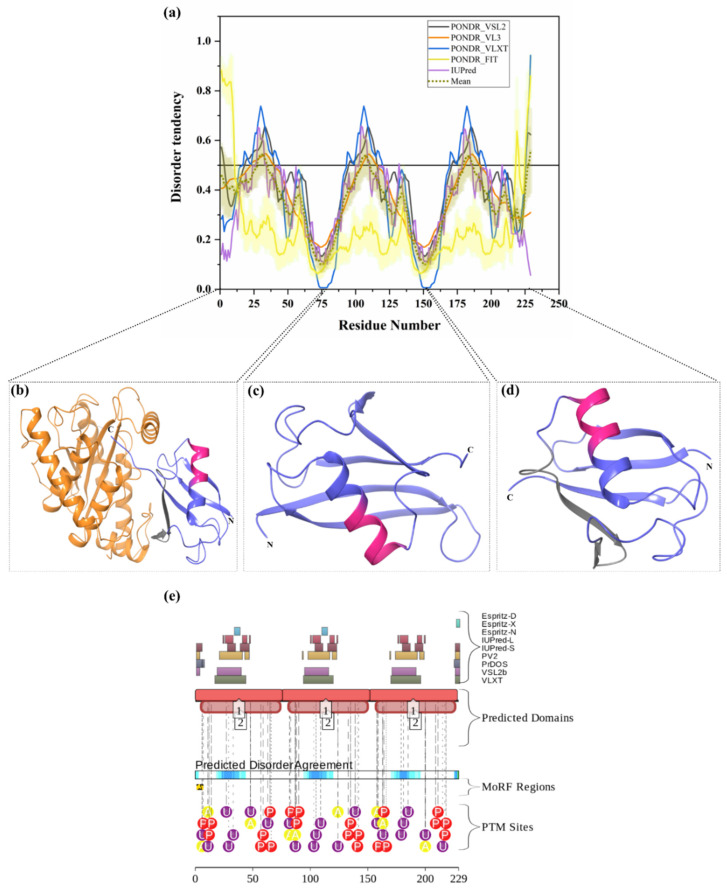
Analysis of intrinsic disorder predisposition in polyubiquitin-B (UBB) and structural characterization of mature ubiquitins. (**a**) Disorder analysis of human polyubiquitin-B (UniProt ID: P0CG47). (**b**) Crystal structure of chain B of Polyubiquitin-B (PDB ID: 6FDK). (**c**) Crystal structure of Chain D of Polyubiquitin-B (PDB ID: 6BYH). (**d**) Crystal structure of Chain A of Polyubiquitin-B (PDB ID: 4XOF). In Plot (**a**), the disorder profile obtained forms a set of disorder predictors such as PONDR^®^ VSL2, PONDR^®^ VL3, PONDR^®^ VLXT, PONDR^®^ FIT and IUPred, represented by black, orange, blue, yellow, and purple curves respectively. Mean disorder profile, which was calculated from average of five predictor-specific per-residue disorder profiles, is shown in olive color. Predicted disorder scores above 0.5 are considered as disordered residues/regions. The light-olive shadow around the mean curve represents the error distribution for the mean. The light-yellow shadow around the PONDR^®^ FIT curve shows the error distribution for PONDR^®^ FIT. In Plot (**b**), a structure of Ub protein (faded blue) in complex with *Chlamydia trachomatis* effector protein Cdu1 (orange color) is represented. In Plots (**b**), (**c**), and (**d**), Ub is shown in faded blue color, and disordered residues are shown in salmon pink color. The position of MoRFCHiBi_Web-server-identified MoRFs (residues 40–50, shown in PDB ID:6FDK, and residues 192–202, shown in PDB ID: 4XOF) are represented by grey color. (**e**) Functional disorder profile of the UBB protein, using the D2P2 server, is shown.

Two E1 enzymes have been identified in mammals, UBA1 and UBA6 [103], with UBA1 (UniProt ID: P22314) is a 1058-amino-acid-long protein most commonly participating in the Ub activation (first step of ubiquitination) in an ATP-dependent manner [64]. The reduced level of E1 enzyme has been reported in AD and HD [64,104]. Furthermore, another neurodegenerative disorder, SMAX2, was found to occur due to the mutation in UBA1 gene [21,105]. Here, according to our disorder analysis, the mean percent of predicted intrinsic disorder (PPID) calculated by averaging the outputs of five commonly used disorder predictors, such as PONDR^®^ VLXT, PONDR^®^ VSL2, PONDR^®^ VL3, PONDR^®^ FIT, and IUPred, was 9.74% (Table 2). Bhowmick et al. reported the presence of structural disorder in the human ubiquitinating enzyme, where they found 5.97% of average percentage of disordered residues in two E1 enzymes (UBA1 & UBA6) by IUPRED [58]. Our IUPRED analysis represents PPID of 8.13% for UBA1, which indicates UBA1 is more disordered than UBA6 enzyme. UBA1 is a multi-domain enzyme containing four domains important for interactions with its partners. The N-terminal half of UBA1 consists of an inactive adenylation domain (IAD) (residues 1–439) that surrounds the first catalytic cysteine half-domain (FCCD) (residues 204–309). We found one IDPR (residues 346–358) in IAD domain (Figure 4(a1)). The C-terminal half of UBA1 consists of an active adenylation domain (AAD) (residues 440–950) that surrounds the second catalytic cysteine half-domain (SCCD) (residues 626–891). One IDPR (residues 813–834) was found through our analysis in AAD and SCCD (Figure 4(a1)). The reactive cysteine residue (C632) is present in SCCD that binds ubiquitin, and our analysis shows that this residue (C632) is located within the ordered region of UBA1 protein. The ubiquitin fold domain (UFD) (residues 951–1058) is present at the C-terminal region of UBA1, which allows this protein to recognize and recruit E2 enzymes [64]. Only one short IDPR (residues 1018–1026) was found in this domain, as shown in Figure 4(a1). In addition, a MoRF region is predicted at the C-terminus of UBA1 by two predictors, MoRFCHiBi_Web (residues 1048–1057) and MoRFpred (residues1051–1058). These IDPRs (mean of five IDP predictors) and MoRFs (MoRFCHiBi_Web) are mapped in Figure 4(a1). Only two crystal structures for UBA1 are available in PDB (PDB ID: 6DC6 (residues 49–1058) [106] and 4P22 (residues 1–439) [107]). However, the N-terminal region containing residues 1–48 is missing in both the available crystal structures. Interestingly, according to our intrinsic disorder analysis, this region was found to be disordered. Furthermore, one disorder-based interaction site was also identified at the N-terminus by three predictors, MoRFCHiBi_Web (residues 1–13), MoRFpred (residues 5–12), and ANCHOR (residues 1–16). Some other IDPRs (residues 346–358, 618–623, 813–834) are represented in available crystal structures (Figure 5(a1)) and identified MoRF regions are listed in Table 3. According to evaluation of PTMs by D2P2, which is represented in Figure 4(a2), UBA1 has 41 phosphorylation sites, of which 25 are located in IDPRs, 10 acetylation sites, of which 5 lie in the IDPRs, and 29 ubiquitylation sites, of which 12 are located in the IDPRs. These results signify the significant role of intrinsic disorder in PTMs of E1 enzyme.

#### 3.1.2. Intrinsic Disorder in Ubiquitin-Conjugating Enzyme (E2 Enzyme)

All E2 enzymes interact with one E1 and one or more E3 enzymes. Humans have around 40 E2 enzymes, which are involved in transfer of Ub or ubiquitin-like proteins such as SUMO and NEDD8 [108]. E2 enzyme has two main functions: transfer of Ub from thioester to a thiol group (trans thiolation) and transfer of Ub from thioester to amino group (aminolysis) [108]. It has one catalytic domain that is made up of ~150 amino acids. Here, the thioester bond is formed between E2 cysteine and the C-terminus of Ub. This domain has four α-helices and four-stranded β-sheets (see Figure 4(b1)). There is also an E3 binding domain that is made up of several loops. Commonly, E2 transfers its thioester-linked Ub to cysteine in the active site of HECT-type E3 ligase in thiolation reactions. Human E2 enzymes seem to show a propensity to transfer Ub to free lysine of RING-type E3 ligases in aminolysis reactions. However, there are a few exceptions [109,110], and Stewart et al., in 2016, reviewed some fundamentally different intrinsic activities of several E2 enzymes. Maximal E2 activity occurs only in the presence of E3 [111]. Most E2 enzymes have short N- and C-terminal extensions that often contain intrinsically disordered regions [112]. Our intrinsic disorder analysis of the UBE2R2 E2 enzyme (UniProt ID: Q712K3) has also predicted the residues at N-terminal (1–23) and C-terminal (198–238) to be disordered (see Figure 4b). Along with these, two short stretches of residues 102–113 and 153–162 also fall in the disordered regions. In total, the disordered regions account for 37.39%, as calculated by mean PPID. Previous studies reported the presence of structural disorder, with 17.74% of the average percentage of disordered residues in 29 E2 enzymes by IUPred [58]. Our IUPRED analysis of UBE2R2 represents PPID of 24.79%. Furthermore, the E2 enzyme also showed disorder binding residues in the C-terminal region, which are predicted by all MoRF predictors (Table 3). MoRFCHiBi_Web server predicted three short MoRF regions (residues 166–170, 203–217, 219–226) at the C-terminal region. DISOPRED3 and MoRFpred also predicted few MoRF residues (1–6 and 11–19, respectively) at the N-terminal region. The recently crystallized structure of E2 enzyme of 1.5 Å resolution has four long and four short helices, forming 33% helical and 6 β-strands constituting a 12% β-sheet structure [113,114]. The unstructured C-terminal also suggested to be disordered, which is in correlation with our disorder analysis. For example, Cdc34 is an E2 protein whose catalytic domain is similar to other E2 proteins, but the acidic C-terminal region (66 amino acids) is disordered, which interacts with Ub in complex. Removal of this interaction leaves Ub free for transfer [113,114]. According to the prediction from D2P2 (Figure 4(b2)), E2 enzyme has five phosphorylations, with all of them in IDPRs, and eight ubiquitylation sites, of which seven are located in the IDPR region. This disordered-region-based analysis of E2 enzyme would be helpful to deeply understand the functioning of this enzyme in the cell.

#### 3.1.3. Intrinsic Disorder in Ubiquitin Ligase (E3 Enzyme)

E3 ligases have been grouped into the RING (really interesting new gene), the HECT (homologous to the E6AP carboxyl terminus), and the RBR (RING between RING) types [115]. RING E3s have a RING or U-box catalytic domain that directly transfers Ub to the target protein. This class of E3s has around six hundred predicted members. They possess a catalytic domain and a substrate-recruiting module, which can be present in a single polypeptide or in different subunits of the multicomponent E3 complex. To understand how E2 stimulates transfer of Ub from E2, crystal structures of two E3 ligases (RNF4 and BIRC7), when they were bound to E2–Ub complex, were studied [116,117]. RING E3 transfers Ub through aminolysis. It alters thioester linkage between the cysteine in the C-terminus of Ub and the cysteine in the active site of E2, thus hydrolyzing the bond. The RING domain is responsible for both binding E2 and stimulating transfer of Ub. It can exist either in the form of a monomer or a dimer. It adopts a cross-brace structure with two Zn^2+^ ions [118]. A subset of E3 ligases called the cullin RING ligases have three subunits: the cullin protein, which acts as the scaffold protein, the RING box protein, which contains the RING domain, and the F-box protein, which binds the substrate. The RING domain helps in positioning Ub on E2 in the correct orientation. A conserved asparagine residue (Asn77) has been proposed to stabilize the oxyanion intermediate [119]. Some RING E3s have additional structural domains, which have been reviewed by Berndson and Wolberger in 2014 [120]. HECT E3 ligases participate in two different reactions: transfer of Ub from Cys in the active site of E2 to Cys in the HECT domain, and HECT-Ub thioester is attacked by substrate lysine. The HECT domain has an N-lobe to bind E2 and a C-lobe that contains active site cysteine. Multiple configurations are made possible via a flexible tether between these two lobes. A conformational change, which is required to bring both the lobes together for successful transfer of Ub to substrate, was also revealed [121]. Similar to E2 enzyme, STUB1 E3 enzyme (Uniprot ID: Q9UNE7) is characterized by a mean PPID of 37.95%. Bhowmick et al. reported the presence of structural disorder in different human E3 ubiquitin ligase families, where they found 20.03% of the average percentage of disordered residues by IUPRED [58]. Furthermore, four disorder-based binding regions (residues 163–169, 198–204, 206–214, 230–239) were detected in this protein as per ANCHOR server (see Table 3). However, not a single MoRF was detected by MoRFCHiBi_Web, MoRFpred, or DISOPRED3. The MoRFs identified by ANCHOR do not contains any region at the N- or C-terminal and are mainly located at the middle region, signifying the role of middle-disordered regions of the E3 enzyme in molecular recognition. A long stretch of 35 residues (1–35) at N-terminal and residues 143–203 are predicted to be disordered, and another long IDPR is predicted by several algorithms in the middle of the protein (residues 147–227) (see Figure 4c). The D2P2 analysis (Figure 4(c2)) recognized several PTMs in disordered regions of E3 enzyme, such as 10 phosphorylation sites, out of which eight lie in IDPRs, one ubiquitylation site lies in IDPRs, and one nitrosylation site lies in IDPRs. For the E3 enzyme, a truncated structure for residues 21–154 is available in PDB (PDB ID: 4KBQ), where initial three residues are missing and other residues lying in disorder regions are mapped (see Figure 4(c1)).

#### 3.1.4. Intrinsic Disorder in Polyubiquitin-B (UBB)

Ubiquitin (Ub) is a 76-amino-acid-long, highly conserved protein expressed in all eukaryotic cells. Ub is encoded by four different genes, where *UBA52* and *RPS27A* code for a single copy of Ub fused to the ribosomal proteins L40 and S27a, respectively, and where a polyubiquitin precursor with exact head-to-tail Ub repeats is encoded by the *UBB* and *UBC* genes, with the corresponding products, polyubiquitin-B and polyubiquitin-C, containing three and six Ub chains, respectively. Mature Ub exists either as a free protein or as a conjugated form that is covalently bound to various intracellular proteins, typically for their degradation through 26S proteasome [15,71]. However, the Ub conjugation to other cellular proteins controls numerous eukaryotic cell functions [122]. Importantly, alterations in UPS have been observed in various types of human cancers and neurodegenerative diseases. Ubiquitin must be supplied in an adequate amount and in a timely manner for conjugation to a variety of proteins. It has been reported that the growth of cancer cells requires ubiquitin, and its downregulation inhibits the ubiquitination of multiple proteins associated with oncogenic pathways [123]. As a result, uncontrolled UPS plays a crucial role in several cellular processes related to tumorigenesis [123]. Increased levels of ubiquitin with enhanced cellular proliferation and stress have been observed in many types of cancer cells [123,124]. Interestingly, protein aggregates associated with familial and sporadic AD often contain proteins other than those, which are generally linked with diseases. One of these proteins is a frameshift form of ubiquitin, UBB+1. UBB+1 is produced by molecular misreading of a wild-type ubiquitin gene, and the presence of this Ub form has been allied with several disorders. UBB+1 accumulation leads to aberrant UPS system activity, which increases the aggregation of toxic proteins leading to cell death [125]. Accumulation of UBB+1 has been reported in several neuronal diseases, such as AD, Pick’s disease, and PolyQ diseases (including HD), as well as non-neuronal tissue diseases [125]. More specifically, UBB+1 accumulation has been reported in the neuritic plaques and neurofibrillary tangles of AD patients [126]. In this study, based on its overall disorder content, UBB (UniProt ID: P0CG47) is classified as a least-disordered or highly ordered protein. The mean PPID for UBB was found to be 10.04% (Table 2). Total 21 residues (28–34, 104–110, and 180–186) in the full-length UBB (229 residues) are predicted to be disordered (see Figure 5a). Furthermore, two predictors, MoRFCHiBi_Web (residues 40–50, 116–122, 192–202) and MoRFpred (residues 221–228), have predicted MoRF residues (Table 3). Similarly, D2P2 has also predicted least disorder and MoRF residues (Figure 5e). PTM analysis by D2P2 in Figure 5e displays 21 phosphorylation sites, of which 1 lies in IDPRs, 9 acetylation sites, of which 5 are in IDPRs, and 21 ubiquitylation sites, of which 14 lie in IDPRs. Multiple crystal structures are available for human Ub in PDB. Figure 5b–d represents three illustrative examples of these structures, where the position of predicted IDPR and MoRF regions are also shown.

#### 3.1.5. Intrinsic Disorder in Ubiquilin 1 (UBQLN1)

**Figure 6:**
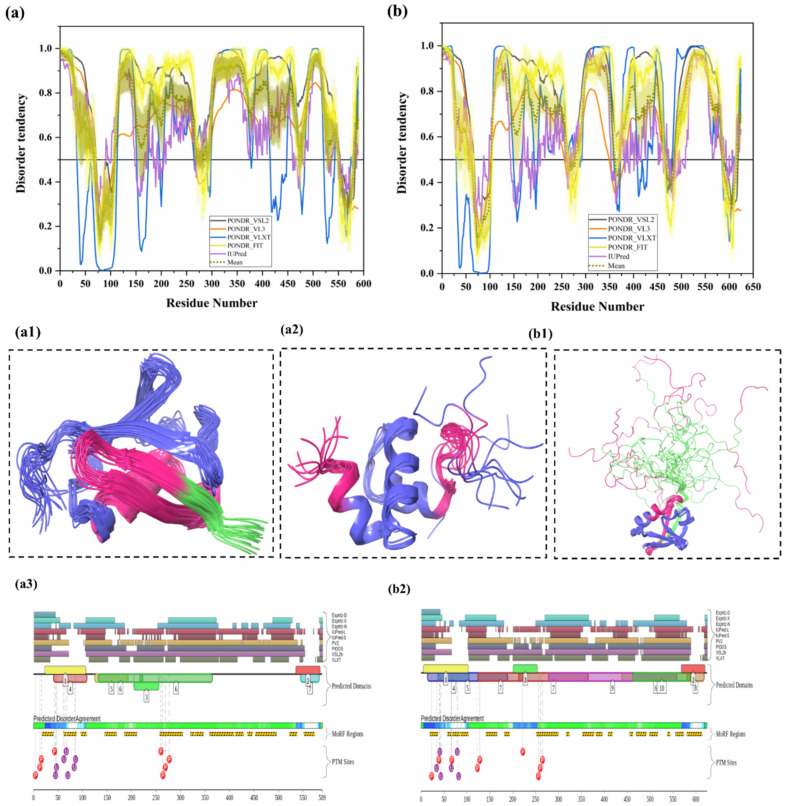
Intrinsic disorder predisposition and structural characterization of human UBQLN1 and UBQLN2. (**a**) Disorder profile of human UBQLN1 (UniProt ID: Q9UMX0). (**b**) Disorder profile of UBQLN2 (UniProt ID: Q9UHD9) (**a1**) NMR solution structure of the N-terminal UBL domain (residues 34–112) of UBQLN1 (PDB ID: 2KLC). (**a2**) NMR solution structure of the C-terminal UBA domain (residues 541–586) of UBQLN1 (PDB ID: 2JY5). (**b1**) NMR solution structure of the N-terminal UBL domain (residues 1–103) of human UBQLN2 (PDB ID: 1J8C). In Plots (**a**) and (**b**), intrinsic disorder profiles generated by disorder predictors, such as PONDR^®^ VSL2, PONDR^®^ VL3, PONDR^®^ VLXT, PONDR^®^ FIT and IUPred, are shown by black, orange, blue, yellow, and purple curves respectively. Mean disorder profile is calculated from the average of five predictor-specific per-residue disorder profiles, represented by the olive color curve. Predicted disorder scores above 0.5 are considered disordered residues/regions. The light-olive shadow around the mean curve represents error distribution for mean. The light-yellow shadow around PONDR^®^ FIT curve shows error distribution for that predictor. In (**a1**,**a2**,**b1**), UBQLN 1 and 2 are represented by faded blue color, and disordered residues are shown in salmon pink color. The position of the MoRF region (residues 34–39) recognized by the MoRFCHiBi_web server in UBQLN1 is represented in (PDB ID: 2KLC), and MoRF region (residues 10–38) in UBQLN2 are represented in (PDB ID: 1J8C) as MoRFs lying in the IDP region by faded green color. (**a3**,**b2**) Functional disorder profile of UBQLN1 and UBQLN2 proteins, respectively, using the D2P2 server, is shown, depicting PTM sites.

Ubiquilin 1 (UBQLN1) (UniProt ID: Q9UMX0) is a 589-residue-long extraproteasomal ubiquitin receptor [127] that plays a crucial role in the regulation of the protein quality control system [6]. Its structure consists of an N-terminal UBL domain (residues 37–111) and a C-terminal Ub-associated (UBA) domain (residues 546–586). Importantly, from our analysis, both UBL and UBA domains were found to be highly disordered and contain many disorder-based binding sites (see Figure 6a,(a1–3)). The UBA domain is identified in ubiquitination-linked proteins, such as E2 and E3 enzymes. Interestingly, ubiquilin interacts, via its UBA domain, more efficiently with poly-Ub chain than with mono-Ub. Ko et al. have shown that ubiquilin interacts especially with the poly-Ub chains of ubiquitylated proteins via the UBA domain and with the subunit of 19S proteasome via the UBL domain [128]. It was also reported that the absence of UBQLN1 is associated with the destruction of protein synthesis and cell cycle arrest [129]. A study has shown that UBQLN1 is essential for the transport of mislocalized mitochondrial proteins to proteasome for their degradation [130]. For proteasomal degradation, the central portion of UBQLN1 is crucial to bind at the hydrophobic domains of mitochondrial proteins [129]. Additionally, in response to myocardial ischemia/reperfusion injury, UBQLN1 plays a significant role in cardiac ubiquitination-proteasome coupling [127]. In approximately 50% of human lung adenocarcinoma cases, UBQLN1 has been reported to be lost or under-expressed [131]. UBQLN1 is also known to regulate the activity and expression of Insulin-like growth factor-1 receptor (IGF1R), a receptor that regulates growth, proliferation, and survival [131]. As a result, UBQLN1 is associated with the pathophysiology of cancer and neurodegenerative diseases [6]. UBQLN1 polymorphism substantially increases the risk of AD, possibly due to its induction of alternative splicing in the brain. UBQLN1 induces Aβ production by affecting APP processing and trafficking. It also regulates the activity of γ-secretase complex by regulating presenilin 1 endoproteolysis within the γ-secretase complex, a protease that cleaves APP and generates Aβ peptide, which is responsible for AD pathogenesis [6]. Furthermore, UBQLN1 also controls the level of β-secretase, a rate-limiting enzyme in the production of Aβ peptides. Therefore, the reduced UBQLN1 level in AD brain may result in perturbed processing of APP and Aβ generation [6]. UBQLN1 acts as a molecular chaperone for APP by binding and preventing its aggregation, and a reduced level of UBQLN1 was found in the brains of AD patients [67]. Several studies have also reported that UBQLN-family proteins are linked to the pathogenesis of PolyQ diseases. Studies in cellular and animal HD models have reported that UBQLN1 suppresses PolyQ-induced protein aggregation and toxicity. Involvement of UBQLN1 in multiple diseases may be due to its highly disordered nature, since this protein is one of the most disordered proteins in a set of UPS-related proteins analyzed in this study. In fact, in our analysis, UBQLN1 was found to be highly disordered, with a mean PPID of 87.10% (Table 2). Figure 6a shows that all five predictors have predicted the presence of long IDPRs in this protein. There are also multiple short as well as long disorder-based binding regions in human UBQLN1 (Table 3). Despite high levels of predicted disorder, NMR solution structures were determined for the comparatively ordered N- and C-terminal regions of human UBQLN1 (Figure 6(a1,a2)). According to D2P2 (Figure 6(a3)), UBQLN1 has eight phosphorylation sites and four ubiquitylation sites, and all of them are located in the IDPR region of this protein. However, many PTMs sites are located at the UBL domain of the UBQLN1 protein. Since UBQLN1 has multiple interactions and functions, results from our analysis (presence of multiple IDPRs and disorder-based binding sites) signify the central role of intrinsic disorder in protein–protein interaction and protein degradation via UPS.

#### 3.1.6. Intrinsic Disorder in Ubiquilin 2 (UBQLN2)

Ubiquilin-2 (UBQLN2) (UniProt ID: Q9UHD9) is a 624-residue-long protein present in cytosol, which is mostly expressed in the brain, liver, spleen, pancreas, heart, and other tissues [6,26]. Like UBQLN1, UBQLN2 is also actively involved in misfolded protein degradation via the ubiquitin-proteasome system [26]. In addition, it also plays an important role in the regulation of the progression of the cell cycle and cellular signaling [69]. Polyubiquitinated proteins that underwent a three-step enzymatic cascade are recognized by the UBL domain of UBQLN2 and transported for degradation to the S5a/PSMD4 receptor of the 26S proteasome [132]. UBQLN2 consists of an N-terminal ubiquitin-like domain (UBL) domain (residues 33–103), which interacts with the proteasome, and a C-terminal ubiquitin-associated (UBA) domain (residues 582–624), which is crucial for the UPS activity. Figure 6b,(b1,b2) shows the presence of significant intrinsic disorder and MoRFs in both domains (UBL and UBA) of UBQLN2. Additionally, UBQLN2 contains one proline-rich repeat domain containing 12 PXX repeats (490–535 AA) involved in protein–protein interactions and four stress-induced protein 1 (STI-1)-like motifs present in regions 178–247 and 379–462, which are responsible for the UBQLN2 interaction with autophagy mediators and HSPs [26]. Interestingly, our ANCHOR-based MoRF analysis found the presence of proline-rich repeat domain and STI-1 motifs in disorder-based binding regions (Table 3). *UBQLN2* gene mutations were reported in frontotemporal dementia (FTD) and amyotrophic lateral sclerosis (ALS). Abnormal UBQLN2 inclusions have been observed in the cytosol of degenerating motor neurons of ALS patients [26]. Furthermore, the disturbance of autophagic and proteasomal protein degradation was reported in ALS-linked mutations in UBQLN2 [133]. Furthermore, ALS-linked mutations in this protein are also linked to neuroinflammation, the formation of stress granules (SGs), and dysfunction of autophagy [26]. Hjerpe et al. reported that mutations in UBQLN2 are associated with defective chaperone binding, impaired aggregate clearance and cognitive deficits in mice and neurodegeneration in humans [134]. Interestingly, numerous mutations were found in the PXX domain of UBQLN2, and these mutations have been reported to provoke impairments in autophagy and 26S proteasome [26]. Studies in neuronal cells have reported that dysregulation of UBQLN2 in neurons may activate NF-κB and cytosolic TDP-43 aggregation [135]. Similar to UBQLN1, UBQLN2 is also predicted to be highly disordered (Figure 6b), with a mean PPID of 80.93%. D2P2 server-based analysis (Figure 6(b2)) provides a further illustration of the highly disordered nature of this protein and shows that UBQLN2 is heavily decorated by multiple PTMs and includes a very large number of MoRFs. According to D2P2 (Figure 6(b2)), UBQLN2 has nine phosphorylation sites located in the IDPRs, and six ubiquitylation sites out of which four lie in IDPR regions. Solution NMR structure was determined for the N-terminal region of human UBQLN2 (residues 1–103) containing UBL domain. Figure 6(b1) shows that this region is characterized by high structural dynamics and conformational flexibility.

#### 3.1.7. Intrinsic Disorder in Ubiquitin C-Terminal Hydrolase Isozyme L1 (UCHL1)

**Figure 7:**
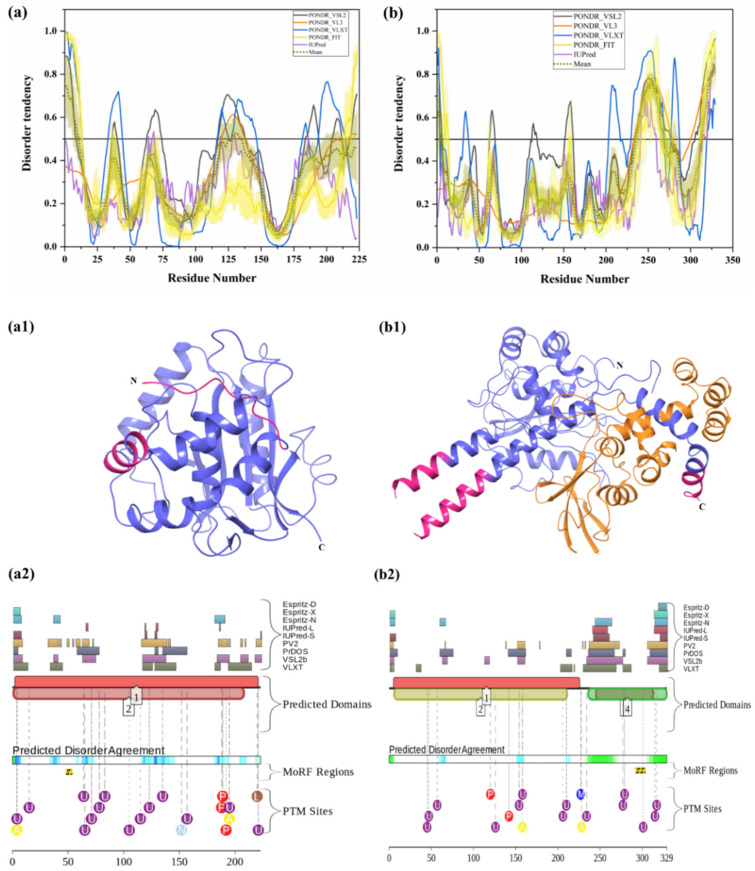
**Intrinsic disorder predisposition and structural characterization of UCHL1 and UCHL5.** (**a**) Disorder profile of human UCHL1 (UniProt ID: P09936) (**b**) Disorder profile of human UCHL5 (UniProt ID: Q9Y5K5) (**a1**) Crystal structure of UCHL1 (PDB ID: 4JKJ). (**b1**) Crystal structure of UCHL5 (PDB ID: 4UEL). In (**a**,**b**), intrinsic disorder profiles obtained from disorder predictors, such as PONDR^®^ VSL2, PONDR^®^ VL3, PONDR^®^ VLXT, PONDR^®^ FIT and IUPred, are depicted by black, orange, blue, yellow, and purple lines respectively. Mean disorder profile, calculated from the average of five predictor-specific per-residue disorder profiles, is represented by the olive color. Predicted disorder scores above 0.5 are considered disordered residues/regions. The light-olive shadow around the mean curve represents the error distribution for the mean. The light-yellow shadow around the PONDR^®^ FIT curve shows the error distribution for PONDR^®^ FIT. In (**a1**,**b1**), UCHL1 and UCHL5 are represented by faded blue color; in Plot (**b1**), the DEUBAD domain of the RPN13 protein and Ub in complex with UCHL5 are shown in orange color. Disordered residues are shown in salmon pink color. In (**a2**,**b2**), the functional disorder profile of UCHL1 and UCHL5 proteins using the D2P2 server have been shown.

UCHL1 (UniProt ID: P09936) is a small, 223-residue-long protein, which is highly abundant in the brain (it is estimated that UCHL1 accounts for 1%–5% of total neuronal protein) and is normally expressed exclusively in neurons and testis [31,70]. UCHL1 catalyzes hydrolysis of C-terminal ubiquityl esters and amides. It is a thiol protease that recognizes peptide bonds at the C-terminal glycine of Ub [136]. The overall structure of this protein (see Figure 7(a1)) resembles a structure typical for the papain family of cysteine proteases, with two lobes: one having five helices and the other having two helices. The identified disordered residues from our analysis are shown in salmon pink color. An active site consisting of a Cys, His, and Asp triad is situated between the two lobes. UCHL1 negatively regulates cytokines and induces NF-κB and STAT1 signaling [137]. Oxidation of a few residues in UCHL1 has been associated with AD. Mutations leading to increased activity of this enzyme have been associated with preserving cognitive functions in AD patients [138]. UCHL1 is essential for the maintenance of axonal integrity, and its dysfunction is associated with neurodegenerative disease [31]. Furthermore, UCHL1 downregulation has been observed in idiopathic AD, as well as in PD brains [139]. I93M mutation in UCHL1 occurs in four out of seven family members who developed PD. According to our analysis, this mutation is located in ordered regions of this protein (Figure 7a). Previous reports suggest that this mutation inhibits α-synuclein degradation via 26S proteasome [31,32]. In-vitro studies have described destabilization of the 3D structure of UCHL1 after deletion of a few amino acids from either the N- or C-terminus, which leads to the partial unfolding of this protein and toxic gain-of-function [31]. The regions consisting of residues 5–10 and 211–216 are involved in interaction with Ub [140]. Although UCHL1 is predicted to have rather low levels of intrinsic disorder, the aforementioned regions related to interaction of this protein with Ub are located within the disordered tails of UCHL1. Furthermore, region 215–220 was predicted to be a disorder-based binding region by the MoRFpred server (see Table 3). According to D2P2 (Figure 7(a2)), UCHL1 has 14 ubiquitylation sites, of which 13 are located in IDPRs. Moreover, it also comprises three phosphorylation sites, two acetylation sites, one nitrosylation site, and one prenylation site, and all of them are located in IDPRs of UCHL1.

#### 3.1.8. Intrinsic Disorder in Ubiquitin C-terminal Hydrolase Isozyme L5 (UCHL5)

UCHL5 (UniProt ID: Q9Y5K5) ia also known as ubiquitin C-terminal hydrolase 37 (UCH37). It is a 329-residue-long DUB that binds to the 19S regulatory subunit of 26S proteasome and deubiquitinates polyubiquitinated proteins. It has a thiol-dependent Ub-specific cysteine protease activity. It is physically associated with a base component of 19S proteasome. It removes poly-Ub chain from the distal end. UCHL5 gets activated by binding of ADRM1, which interacts with the C-terminal tail of UCHL5, a region that is different from the UCH catalytic domain [33,141]. Ub-mediated degradation occurs in the absence of Hedgehog signaling. UCHL5 is a critical regulator of hedgehog signaling [141]. Ge et al. reported lower expression of this enzyme in the brain tissues of glioma patients [142]. In vitro analysis revealed that UCHL5 can inhibit migration and invasion of glioma cells mediated via a downregulation of SNRPF [142]. It was reported that UCHL5 deubiquitinates Tcf3, which helps it to fully activate the Wnt/β–catenin pathway [143]. Furthermore, the region of UCHL5 amino acid residues from 313–329 interacts with proteasomal receptor ADRM1 [144], and, according to our analysis, this ADRM1 binding region of UCHL5 is the part of disorder-based protein binding region (see Table 3). Human UCHL5 is predicted to have more intrinsic disorder than UCHL1 (PPID of 16.11% vs. 7.62%) (compare Figure 7a,b). D2P2 server (Figure 7(b2)) identified several PTMs in UCHL5. These include two phosphorylation sites, of which one is located in the IDPRs, two acetylation sites, of which one is located in the IDPRs, 14 ubiquitylation sites, of which 10 are located in IDPRs, one nitrosylation site in IDPR, and one mono-methylation site. Interestingly, some PTM sites are located in the ADRM1 binding region of this protein. These results signify the important role of intrinsic disorder in UCHL5 for interaction with proteasome receptor ADRM1 and further 26S proteasome-mediated degradation of target proteins.

#### 3.1.9. Intrinsic Disorder in Ubiquitin-Specific-Processing Protease 7 (USP7)

**Figure 8:**
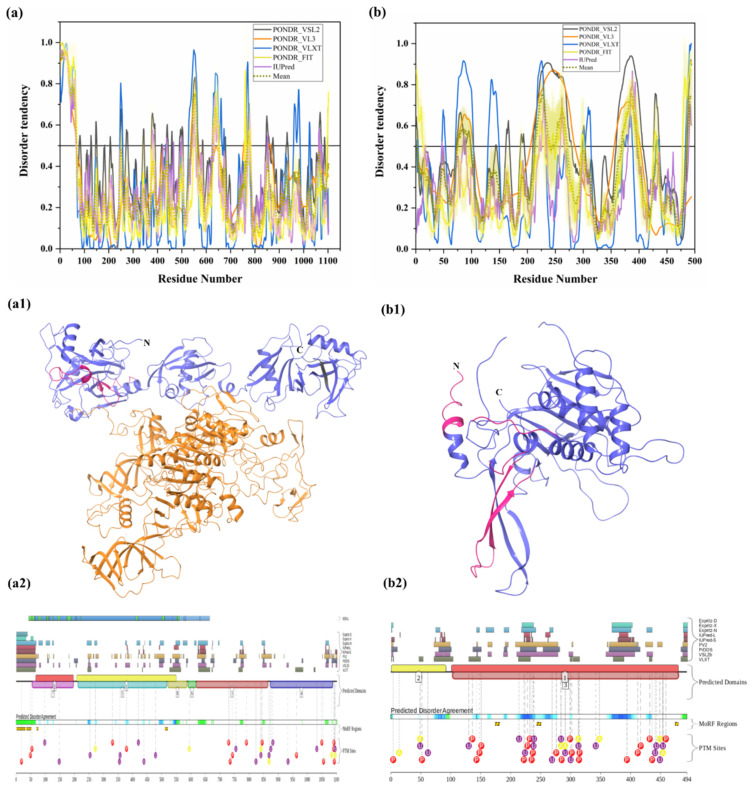
Intrinsic disorder predisposition and structural characterization of human USP7 and USP14. (**a**) Disorder profile of human USP7 (UniProt ID: Q93009). (**b**) Disorder profile of human USP14 (UniProt ID: P54578) (**a1**) crystal structure of USP7 (PDB ID: 4YOC). (**b1**) Crystal structure of USP14 (PDB ID: 4GJQ). In Plots (**a**) and (**b**), disorder profiles generated by sets of disorder predictors such as PONDR^®^ VSL2, PONDR^®^ VL3, PONDR^®^ VLXT, PONDR^®^ FIT, and IUPred are depicted by black, orange, blue, yellow, and purple curves respectively. The mean disorder profile calculated from the average of five predictor-specific per-residue disorder profiles is shown by the olive color curve. Predicted disorder scores above 0.5 are considered disordered residues/regions. The light-olive shadow around mean curve represents the error distribution for the mean. The light-yellow shadow around the PONDR^®^ FIT curve shows the error distribution for PONDR^®^ FIT. In Plots a1 and b1, USP7 and USP14 are represented by faded blue color; in Plot (**a1**), human DNA (cytosine-5)-methyltransferase 1 (DNMT1) complexed with USP7 is shown in orange color. Disordered residues are shown by the salmon pink color. In Plot (**a1**), the positions of MoRFs (residues 1077–1082) predicted by the MoRFCHiBi_web server are shown by grey color in USP7 (PDB ID: 4YOC). (**a2**,**b2**) depict the PTM sites and MoRF regions obtained from the D2P2 server.

USP7 (UniProt ID: Q93009) is also known as ubiquitin carboxyl-terminal hydrolase 7 or herpesvirus-associated ubiquitin-specific protease (HAUSP), and is a member of the USP family of deubiquitylating enzymes; it cleaves ubiquitin from polyubiquitin chains of substrate protein [10,145,146]. Figure 8(a1) represents the crystal structure of USP7 (PDB ID: 4YOC), and IDPRs and MoRFs identified in this study are denoted by salmon pink and grey color, respectively. This 135-kDa cellular protein is associated with numerous cellular processes, such as oncogenesis and tumor suppression, immune functions, DNA dynamics, epigenetic modulations, DNA damage and repair processes, regulation of gene expression and protein function, and host–virus interactions. Dysfunctions in USP7 at different physiological conditions lead to the development of various pathological conditions, such as cancer, immune dysfunction, metabolic diseases, and neurological pathologies. USP7 is mainly recognized in cancers and virus-associated host–pathogen interactions [10]. The full-length USP7 consists of 1102 amino acids [147] and has several functional domains, including poly Q stretch (amino acid 4–10), tumor necrosis factor receptor-associated factor (TRAF)-like domain, C-terminal domain (CTD), and middle catalytic (CAT) domain [10,147]. The presence of poly-Q repeat in HSP7 specifies that it may have a link to the neurodegenerative disorders associated with the poly-Q expansion [10]. TRAF domain (amino acids 62–208) is required for protein–protein interactions and also plays an important role in nuclear localization of USP7 [147,148]. The TCAT domain (amino acids 208–560) is crucial for the catalytic activity. Some residues from this domain are located in the disorder-based binding regions (Table 3). This catalytic domain mediates ubiquitination and deubiquitination of substrate proteins. CTD (amino acids 560–1102) consists of five ubiquitin-like (UBL) folds that enable protein–protein interactions with other proteins, such as ICP0, ataxin-1, DNA (cytosine-5-)-methyltransferase 1 (DNMT1), and ubiquitin-like PHD and RING finger domain-containing protein 1 (UHRF1). MDM2 and p53 also interact with USP7 via CTD, which serves as a second site of interaction [10,147,149,150]. Interestingly, our analysis found MoRF regions (Table 3) at CTD of USP7 using three different servers: MoRFCHiBi_Web (residues 1077–1082 and 1090–1102), MoRFpred (residues 1094–1099), and DISOPRED3 (residues 1056–1061 and 1084–1093). In various pathological conditions, variation in the USP7 expression level has been observed in different organs. However, not a single mutation has been reported to date in the USP7 gene [10]. Being one of the longest proteins in the set analyzed in this study, USP7 is characterized by relatively low disorder content (see Figure 8a). In fact, the mean PPID of this protein is 11.62%. However, Table 3 and Figure 8(a2) show that human USP7 has several MoRFs and multiple PTM sites including 16 phosphorylation sites, of which nine are located in the IDPRs, six acetylation sites, of which four are located in the IDPRs, and 17 ubiquitylation sites, of which nine are located in the IDPRs. These results suggest the functional importance of intrinsic disorder in this protein.

#### 3.1.10. Intrinsic Disorder in Ubiquitin Carboxyl-Terminal Hydrolase 14 (USP14)

USP14 (UniProt ID: P54578), is a proteasome-associated deubiquitinating enzyme, which is unique among known UBP enzymes [35,151]. Figure 8(b1) is the crystal structure of USP14 (PDB ID: 4GJQ), where IDPRs found in our analysis are represented in salmon pink color. It plays an important role in the development of synapses [152]. Furthermore, it is crucial for the degradation of ubiquitinated proteins, since proteasome activation occurs when the polyubiquitin chain binds to USP14, which further degrades substrate protein [153]. This reduces the degradation of toxic/pathogenic misfolded proteins by the proteasome, and studies show that USP14 inhibition enhances the degradation of toxic proteins associated with neurodegenerative diseases, such as AD, ALS, PD, HD, etc. [36]. Therefore, inhibitors of USP14 may provide good therapeutics for protein aggregation diseases. Full-length human USP14 consists of 494 amino acids, with ubiquitin-like (UBL) domain (9 kDa) (residues 1–90) at the N-terminus followed by the catalytic domain (45 kDa) (residues 91–494) [35], which is vital for the catalytic activity of USP14, whereas the C-terminal domain facilitates protein–protein interaction [35]. One disorder-based binding region (residues 66–75) was identified by DISOPRED3 in the UBL domain (Table 3). In addition, many PTM sites are also located in the C-terminal catalytic domain (Figure 8(b2)). The interaction of USP14 with the 19S regulatory particle of 26S proteasome through its UBL domain stimulates DUB’s catalytic activity one-hundred-fold [154]. It directly interacts with the ATPase ring of the proteasome, which leads to the conformation changes in the 19S proteasome that allow proper substrate interaction. It negatively regulates proteasome activity. Furthermore, it negatively regulates autophagy since its genetic inhibition results in downregulation of autophagic flux. Most USP proteins consist of two ubiquitin binding domains; one for proximal Ub, which cleaves isopeptide linkage between two ubiquitin moieties, and the other for distal Ub. The catalytic core consists of Cys, His, and Asp/Asn residues. There are two Cys-X-X-Cys motifs in human USP14 that bind zinc, which may help in proper folding of the core [155]. Figure 8b shows that USP14 is predicted to have several IDPRs, possessing the relatively high levels of intrinsic disorder with a mean PPID of 23.48%, whereas Figure 8(b2) indicates that some of the IDPRs in this protein are used for protein–protein interactions (see also Table 3) and serve as a site of various PTMs, including 23 phosphorylation sites, of which 19 are located in the IDPRs, eight acetylation sites, of which 3 are located in the IDPRs, and 17 ubiquitylation sites, of which 10 are located in IDPRs.

#### 3.1.11. Intrinsic Disorder in Ataxin-3 (ATXN3)

**Figure 9:**
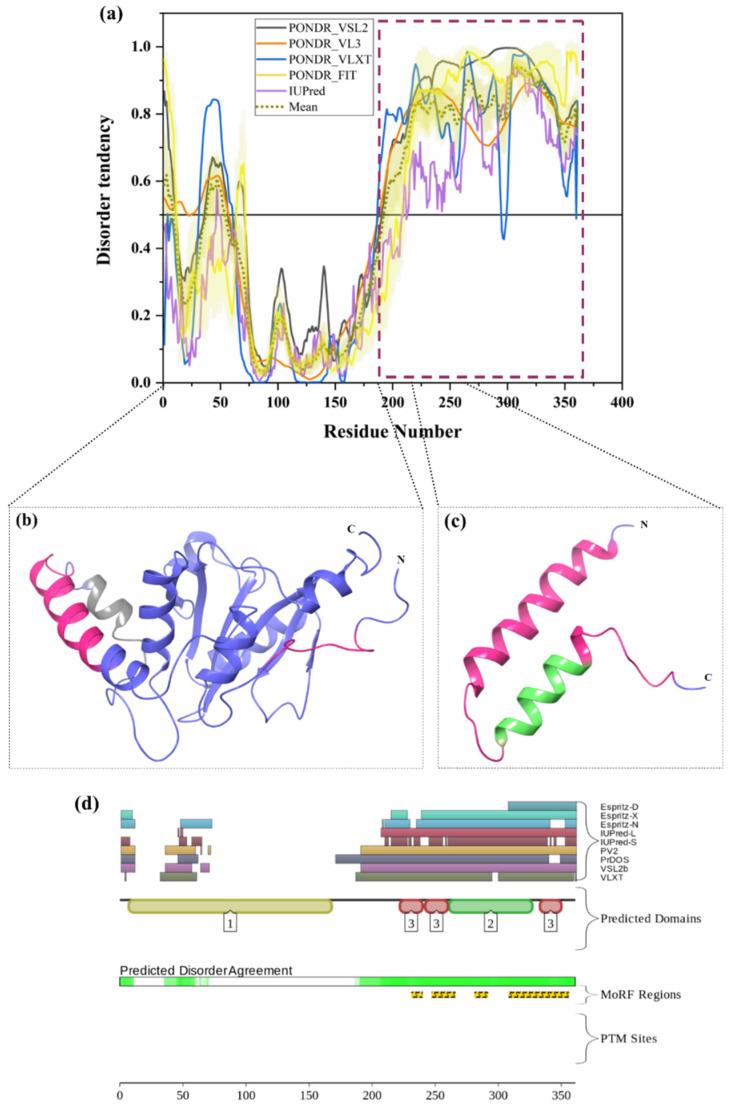
Intrinsic disorder predisposition of ataxin-3. (**a**) Intrinsic disorder profile generated for ataxin-3 (UniProt ID: P54252) by a set of per-residue disorder predictors, such as PONDR^®^ VSL2, PONDR^®^ VL3, PONDR^®^ VLXT, PONDR^®^ FIT, and IUPred. (**b**) Crystal structure of the Josephin domain of ataxin-3 (PDB ID: 2AGA). (**c**) NMR solution structure of the tandem UIM domain of ataxin-3 (PDB ID: 2KLZ). In Plot (**a**), disorder profiles generated by set of disorder predictors, such as PONDR^®^ VSL2, PONDR^®^ VL3, PONDR^®^ VLXT, PONDR^®^ FIT, and IUPred, are depicted by black, orange, blue, yellow, and purple curves respectively. A mean disorder profile calculated from average of five predictor-specific per-residue disorder profile is shown by the olive color curve. Predicted disorder scores above 0.5 are considered as disordered residues/regions. The light-olive shadow around the mean curve represents the error distribution for the mean. The light-yellow shadow around the PONDR^®^ FIT curve shows the error distribution for PONDR^®^ FIT. In Plots (**b**) and (**c**), ataxin-3 is represented by a faded blue color; disordered residues are shown by a salmon pink color. In (**b**), the position of MoRFs (residues 56–65) predicted by the MoRFCHiBi_web server is shown by a gray color (PDB ID: 2AGA). In (**c**), the position of MoRFs (residues 246–255) predicted by the MoRFCHiBi_Web server is represented as MoRFs lying in IDPRs by faded green color (PDB ID:2KLZ). (**d**) The D2P2 server-based functional disorder profile is shown.

ATXN3 (UniProt ID: P54252) is a 42-kDa ubiquitously expressed deubiquitinating enzyme involved in the degradation of misfolded chaperone substrates, transcription, cytoskeleton regulation, and maintenance of protein homeostasis [39,74,156]. Polyglutamine repeat expansion in the unstructured C-terminus of the human ataxin-3 protein leads to Spinocerebellar Ataxia Type 3 (SCA3), an age-related neurodegenerative disease [74]. CAG repeat expansion in the ATXN3 coding region is a crucial molecular defect reported in SCA3. Blount et al reported that ubiquitin-binding site 2 (UbS2) of ATXN3 interacts with Rad23 and prevents its degradation by proteasome [73]. Ataxin-3 is a ubiquitin-specific protease that mainly binds with the long polyubiquitin chains of unwanted proteins through its C-terminal UIMs and cleaves the ubiquitin from polyubiquitin-tagged proteins through its N-terminal ubiquitin protease domain just before they are degraded by 26 S proteasome so that the ubiquitin can be used again. However, reports have shown that ATXN3 shows weak or no activity for the chains with four or fewer ubiquitins [74,157]. Besides the important role of ATXN3 in the degradation of proteins, it has been associated with the regulation of transcriptional process. Interestingly, ATXN3 directly binds to DNA through a leucine zipper motif present in between 223 to 270 amino acids [39]. The structure of Ataxin-3 contains two ubiquitin-binding sites UbS1 (residues 77–78) and UbS2 (residue 87) on the catalytic domain. UbS2 mainly controls normal ATXN3 protein levels and turnover in cells. Next to UbS2, three ubiquitin-interacting motifs (UIM) are present, such as UIM1 (residues 224–243), UIM2 (residues 244–263) and UIM3 (residues 331–349), which bind to ubiquitin chains at least four moieties long [73]. Interestingly, from our analysis, all three UIM are present in disordered regions of ATXN3 (Figure 9a,c,d). Moreover, several disorder-based binding regions are present in three UIMs (Table 3). UIMs function in mediating high-affinity binding of ATXN3 to Ub chains. The IDPRs and MoRFs identified in our analysis are represented in Figure 9b,c on the crystal structure of the Josephin domain of ataxin-3 (PDB ID: 2AGA) and the NMR solution structure of the tandem UIM domain of ataxin-3 (PDB ID: 2KLZ). In addition, UIMs restrict the chain types that can be trimmed by the ATXN3 protein [158]. These UIMs are separated by the polyglutamine (polyQ) domain, which interacts with BECN1 via the deubiquitination of the Lys-402 of BECN1 that stabilizes BECN1 leads to starvation-induced autophagy [159]. The VCP binding site (residues 282–285) is present prior to the polyQ region, which was found to be located in disordered and MoRF regions of this protein [73]. Notably, the Arginine-rich region at the C-terminal of ATXN3 binds the AAA ATPase protein VCP [73]. A nuclear localization signal (NLS) is present in the region from 273 to 286 amino acids, which is also located in disordered and MoRF regions. Residues 1–27 and 237–286 are important for cellular localization, and both the regions are found to be MoRFs (see Table 3). Additionally, ATXN3 also contains six nuclear export signals (NES); among them, residues 77–99 and 141–158 are important NES since they show significant nuclear export activity. The large Josephin domain at the N-terminus of ATXN3 has low isopeptidase activity. UIMs along with the Josephin domain either rescue proteins from degradation or stimulate protein degradation by deubiquitinating protein and maintaining free reusable ubiquitin [39]. Finally, mutation in ATXN3 leads to consequences such as impaired autophagy, compromised axonal transport, mitochondrial dysfunction, transcriptional deregulation, and proteasomal dysfunction [39]. Figure 9a,d shows that the C-terminal half of human ATXN3 is predicted to be highly disordered, possessing several MoRF sites; however, no single PTM site was identified. Importantly, the vast majority of the aforementioned functional regions of this protein are concentrated within this IDPR, clearly indicating the importance of intrinsic disorder for the overall functionality of human ATXN3.

#### 3.1.12. Intrinsic Disorder in Adhesion-Regulating Molecule 1 (ADRM1)

**Figure 10:**
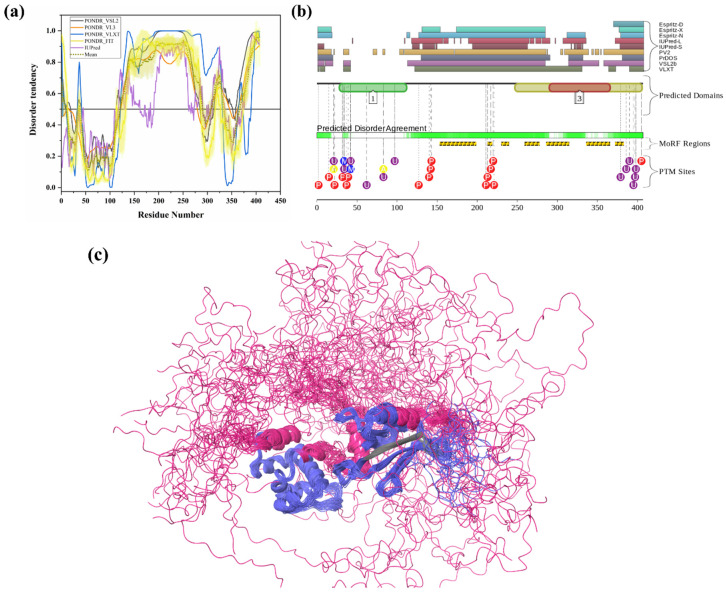
Intrinsic disorder predisposition of ADRM1. (**a**) Intrinsic disorder profile generated for ADRM1 (UniProt ID: Q16186) by a set of per-residue disorder predictors, such as PONDR^®^ VSL2, PONDR^®^ VL3, PONDR^®^ VLXT, PONDR^®^ FIT, and IUPred. (**b**) D2P2 server-based functional disorder profile for ADRM1. (**c**) Solution NMR structure of the ADRM1 with 20 different conformations (PDB ID: 2KR0). In Plot (**a**), disorder profiles generated by sets of disorder predictors, such as PONDR^®^ VSL2, PONDR^®^ VL3, PONDR^®^ VLXT, PONDR^®^ FIT, and IUPred, are depicted by black, orange, blue, yellow, and purple curves, respectively. The mean disorder profile, calculated from the average of five predictor-specific per-residue disorder profiles, is shown by the olive color curve. The predicted disorder score above 0.5 are considered as disordered residues/regions. The light-olive shadow around mean curve represents the error distribution for the mean. The light-yellow shadow around PONDR^®^ FIT curve shows error distribution for PONDR^®^ FIT. In (**c**), ADRM1 is represented by the faded blue color, and disordered residues are shown by the salmon pink color.

ADRM1 (also known as proteasome regulatory particle non-ATPase 13, RPN13; UniProt ID: Q16186) is a 407-residue-long protein, which, in addition to being a component of the 26S proteasome, also has a role in cell adhesion. It is a proteasomal receptor that recognizes K-48-linked poly-Ub chains on the target proteins. It also acts as a receptor for a deubiquitinase, UCHL37. ADRM1 has a Pleckstrin-like a receptor for the ubiquitin domain located at its N-terminal region (residues 22–130), which binds to Ub. From our analysis, this domain is the part of the ordered region in ADRM1 (Figure 10a). Husnjak et al., in 2008, showed that blocking ADRM1 via SiRNA or RA190 triggers plasmacytoid dendritic cell, cytotoxic T lymphocyte, and natural killer cell-mediated lysis of multiple myeloma cells [160]. Jiang et al. demonstrated, in 2017, that levels of ADRM1 are elevated in high-grade ovarian serous carcinoma and serous tubal intraepithelial carcinoma [161]. Importantly, the inhibition of ADRM1 leads to the accumulation of poly-Ub-proteins, triggering apoptosis in cancer cells [161]. The NMR solution structure determined for full-length human ADRM1 (PDB ID: 2KR0) revealed that this protein contains high levels of intrinsic disorder, with the N- and C-terminally located ubiquitin- and UCH37-binding domains (residues 22–130 and 253–407, respectively) being packed against each other when ADRM1 is not incorporated into the proteasome [144]. Figure 10c represents this rather unusual structure, where a large central part of the protein is completely unstructured. In line with these observations, Figure 10a,b shows that the central 150-residue-long region is predicted to be highly disordered but contains multiple PTMs and MoRFs. According to D2P2 (Figure 10b), ADRM1 has 16 phosphorylation sites, of which 15 are located in the IDPRs, 2 acetylation sites in the IDPRs, 12 ubiquitylation sites, of which 9 are in IDPRs, and 2 mono-methylation sites, of which 1 is located in IDPRs. Fascinatingly, ADRM1 is the proteasomal ubiquitin receptor, which plays a vital role in recognition, recruitment, and eventually degradation of protein substrates through 26S proteasome in a well-controlled manner. These processes require interaction with biological partners and the identified dynamic and flexible regions may have crucial roles for protein degradation. Therefore, ADRM1 could be targeted for drug development against cancer as well as neurodegenerative diseases.

#### 3.1.13. Intrinsic Disorder in 26S Proteasome Non-ATPase Regulatory Subunit 2 (PSMD2)

**Figure 11:**
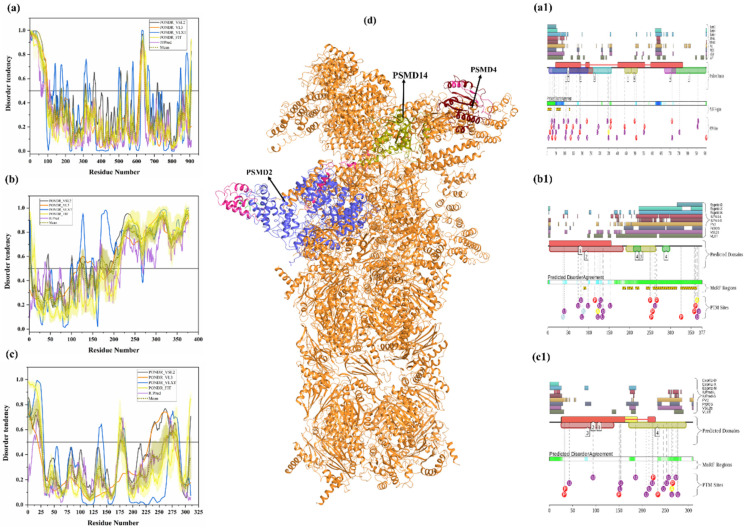
Intrinsic disorder predisposition of human PSMD2 (**a**), PSMD4 (**b**), and PSMD14 (**c**). Plots show the intrinsic disorder profile generated by a set of per-residue disorder predictors, such as PONDR^®^ VSL2, PONDR^®^ VL3, PONDR^®^ VLXT, PONDR^®^ FIT, and IUPred. The corresponding outputs are depicted by black, orange, blue, yellow, and purple curves respectively. Mean disorder profile is calculated from the average of five predictor-specific per-residue disorder profiles and shown by the olive color curve. Predicted disorder scores above 0.5 are considered disordered residues/regions. Light-olive shadow around the mean curve represents the error distribution for the mean. The light-yellow shadow around the PONDR^®^ FIT curve shows the error distribution for that predictor. (**d**) portrays the cryo-EM structures of substrate-engaged human 26S proteasome in seven different conformational states (PDB ID: 6MSB); all three reported proteins PSMD2 (blue), PSMD4 (wine) and PSMD14 (olive) are shown in this complex. D2P2 server-based functional disorder profiles are shown in (**a1**,**b1**,**c1**).

PSMD2 (also known as 26S proteasome regulatory subunit RPN1; UniProt ID: Q13200) is a 100-kDa protein composed of 909 amino acids. PSMD2 may have a role in presenting ubiquitinated substrates to the proteasome. Shi et al., demonstrated, in 2016, that PSMD2 has two binding sites, T1 and T2, in its toroid domain, which interact with Ub, UBL of shuttles, and the UBL domains of DUB and Ubp6, respectively [162]. It has a leucine-rich repeat like domain at its N-terminus, a horseshoe-shaped structure which has a β-sheet at its inner side that interacts with the UBL domains of Rad23 and Dsk2 shuttles and helps in unloading the substrate onto the proteasomal ATPases [163]. Furthermore, the knockdown of PSMD2-suppressed cell proliferation in breast cancer cell lines and also an upregulation of p21 and p27 was seen [164]. Figure 11a shows that, although human PSMD2 is mostly ordered, it contains several long IDPRs, including a 100-residue-long N-terminal region, which is heavily decorated by different PTMs (see Figure 11(a1)), including 17 phosphorylation sites of which 12 are located in the IDPRs, 27 ubiquitylation sites, of which 16 are located in IDPRs, and 1 acetylation site. In addition to PTM sites, the N-terminal region also comprises several disorder-based binding regions (Table 3) which were identified by three different predictors: MoRFCHiBi_Web (residues 1–13, 96–102), MoRFpred (residues 51–62), and ANCHOR (residues 1–30, 35–79). These regions may have a vital role in polyubiquitinated protein recognition on 19S RP of the 26S proteasome.

#### 3.1.14. Intrinsic Disorder in 26S Proteasome Non-ATPase Regulatory Subunit 4 (PSMD4)

PSMD4 is a non-ATPase 19S base component of the 26S proteasome complex (UniProt ID: P55036). Apart from this, it is also present in substantial amounts in free form. This is a 41-kDa protein composed of 377 amino acids. It is also called RPN10 and is responsible for recognizing Ub moieties on proteins. It has an N-terminal von Willebrand factor A domain (VWA) (residues 5–188) and two C-terminal helical UIM (residues 211–230 and 282–301). From our analysis, we observed that the N-terminal VWA domain is located in an ordered region, and the C-terminal UIM is located in disordered regions of PSMD4 (Figure 11b,(b1)). Moreover, several MoRF regions and PTMs are found in the UIM domain (Table 3 and Figure 11(b1)). Mice expressing PSMD4 lacking the UIM domains but having an intact VWA domain died in utero, whereas liver-specific deletion of UIMs resulted in the accumulation of ubiquitinated proteins; yet, these mice lived longer than PSMD4-null mice, suggesting that the VWA domain may act as a facilitator [165]. Jiang et al. demonstrated that knockdown of PSMD4 in hepatocellular carcinoma cell lines suppressed cell proliferation, which could be reversed by overexpressing AKT as PSMD4 promoted PTEN degradation [166]. Recently, Chen et al. found that UIM-2 of PSMD4 is capable of interacting with the UBL domain of UBQLN2 and prefers K11 and K48 Ub linkages in substrates [167]. They also resolved the structure of these interactions [167]. Figure 11b shows that, when not in a complex with the proteasome, a fragment of the PSMD4 protein (residues 196–306) containing two UIMs is characterized by high conformational flexibility (PDB ID: 1YX4) [168]. This is in line with the results of the prediction of intrinsic disorder predisposition of this protein (Figure 11b,(b1)), showing high levels of disorder in the C-terminal part of this protein. Similar to IDPRs in other proteins, this disordered C-terminal region of PSMD4 contains multiple PTMs and disordered binding regions. D2P2 analysis (Figure 11(b1)) predicted the presence of 8 phosphorylation sites in IDPRs, 14 ubiquitylation site, of which 11 are located in IDPRs, 2 acetylation sites in IDPRs, and 2 nitrosylation sites, of which 1 is located in a disordered region. Importantly, regions important for interaction with UBQLN1 (residues 197–262) or binding to Ub (residues 216–220 and 287–291) are all localized within this disordered half, as well as MoRF regions of this protein, indicating the crucial role of intrinsic disorder in PSMD4 function.

#### 3.1.15. Intrinsic Disorder in 26S Proteasome Non-ATPase Regulatory Subunit 14 (PSMD14)

PSMD14 (also called Rpn11; UniProt ID: O00487) is made up of 310 amino acids present in the 19S cap and helps in substrate deubiquitination [169]. It is a zinc-dependent metallopeptidase that belongs to the JAMM family of proteases [170]. It is present in a heterodimeric complex with Rpn8. The cryo-EM structures of substrate-engaged human 26S proteasome in seven different conformational states (PDB ID: 6MSB) are shown in Figure 11d, where PSMD14 is represented in olive color. Downregulation of the PSMD14 gene has been associated with AD [171]. The knockdown of PSMD14 via RNAi has been reported to induce cell cycle arrest, ultimately leading to senescence in carcinoma cell lines [76]. So far, this enzyme has been implicated in cancer by many researchers. In cancer cells, RNAi of PSMD14 decreased proteasome activity and inhibited cell growth. Zhang et al. reported that the human ortholog of PSMD14, POH1, deubiquitinates pro-interleukin-1β, which helps in suppressing inflammasome activity in macrophages of mice [172]. Furthermore, the knockdown of POH1 inhibited tumor progression and induced apoptosis in mitochondria in vitro and RNAi of POH1 achieved similar results in vivo [173]. PSMD14 stabilizes the SNAIL protein by deubiquitination, which is associated with human esophageal squamous cell carcinoma [174]. Similarly, this enzyme has also been associated with breast cancer [38]. In an interesting study by Song et al., it was shown that PSMD14 is overexpressed in multiple myeloma cells, and its pharmacological inhibition helps these cells to overcome their resistance to the proteasome inhibitor bortezomib [175]. PSMD14 is involved in various biological processes, such as programmed cell death, DNA repair, and embryonic cell development and differentiation [38]. Mpr1p, Pad1p N-terminal (MPN) domain (residues 31–166) present at the N-terminal region of the protein is crucial for the functioning of PSMD14, which releases ubiquitin from ubiquitinated proteins [176], and our analysis identified that the MPN domain is located in the ordered region of PSMD14 protein (see Figure 11c,(c1)).

According to the multifactorial disorder analysis, human PSMD14 contains several IDPRs (see Figure 11c). Some of these IDPRs serve as disorder-based binding sites (see Table 3), whereas others are PTM sites (see Figure 11(c1)), which include six phosphorylation sites, of which four are located in IDPRs, 14 ubiquitylation sites, of which 8 are located in IDPRs, and one acetylation site in a disordered region. At the N-terminal region of PSMD14, a short MoRF region identified by three different servers—MoRFCHiBi_Web (residues 1–7), MoRFpred (residues 1–9), and DISOPRED3 (residues 1–12)—indicates participation of its N-terminal region in 26S proteasome-mediated degradation of polyubiquitinated target protein.

## 4. Conclusions

The ubiquitin proteasome system plays a key role in the pathogenesis of various types of cancers and neurodegenerative diseases. The components of UPS actively participate in the process of protein degradation, and any disturbance in this system leads to the occurrence of aforementioned diseases. Our intrinsic disorder and MoRF analysis of disease-associated UPS proteins recognized numerous functional IDPRs and disorder-based binding regions. Using five different IDP prediction tools, we found that UBA1 and UCHL1 are highly ordered; USP14, PSMD14, UCHL5, PSMD2, USP7, and UBB are moderately disordered; and UBQLN1, UBQLN2, ADRM1, PSMD4, ATXN3, STUB1, and UBE2R2 are highly disordered proteins. We also documented multiple post-translational modifications (PTMs) sites in all the proteins except ataxin-3, and most of the identified PTMs are located in the disordered regions of proteins. Since these proteins have to interact with their biological partners for the normal functioning of protein homeostasis, disorder-based binding regions may have an important role due to their flexibility and dynamic nature. For example, the 19S regulatory particle (RP) of the 26S proteasome contains receptors (such as ADRM1 and PSMD4) for interaction with the polyubiquitin chain of the target protein. Interestingly, in this study, these receptors are found to be highly disordered. Moreover, the domains of extraproteasomal polyubiquitin receptors (UBQLN1 and UBQLN2), which are responsible for interaction with proteasome as well as polyubiquitinated proteins for their degradation, are found to be highly disordered. These IDPRs in the components of UPS may have functional roles in maintaining cellular protein homeostasis, normal functioning of UPS, and aberrant UPS functionality in the pathogenesis of the diseases mentioned above. Further studies are required for complete elucidation of the roles of these identified IDPRs and disorder-based binding regions in the pathogenesis of diseases.

## Figures and Tables

**Table 1:** Proteins involved in the ubiquitin proteasomal system (UPS).

Sr. No.	Protein/Gene Name	Length (Amino Acids)	Function in UPS	Altered Function of Protein in UPS	Involvement in Diseases	UniProt ID	References
1	Ubiquitin-like modifier-activating enzyme 1 (UBA1)	1058	Catalyzes first step in ubiquitination. Binds with Ub, activates it and transfers it to E2 enzyme	Reduced level of UBA1 affects UPS-mediated protein degradation, missense mutations in UBA1 gene lead to SMAX2	Neurological disorders such as AD, SMAX2, and HD	P22314	[21,63,64]
2	Ubiquitin-conjugating enzyme E2 R2 (UBE2R2)	238	Catalyzes second step in ubiquitination. Accepts Ub from E1 enzyme and binds with E3 enzyme	Mutation and dysregulation in UBE2R2 affect UPS function	Dysregulation leads to cancer or neurodegenerative diseases	Q712K3	[23,65]
3	E3 ubiquitin-protein ligase enzyme CHIP (STUB1)	303	Catalyzes final step of ubiquitination. Binds with target protein and transfers Ub from E2 enzyme to target protein.	Deregulation of E3 enzyme affects UPS-mediated degradation process.	Deregulation leads to cancer and neurodegenerative diseases such as AD, PD, and HD	Q9UNE7	[23,24,66]
4	Polyubiquitin-B (UBB)	229	Tags target proteins for proteasomal degradation	Frameshift mutation in ubiquitin-B forms UBB+1, which disturbs UPS-mediated protein degradation	UBB+1 accumulation with Aβ in AD and Down’s syndrome	P0CG47	[18,19]
5	Ubiquilin-1 (UBQLN1)	589	Regulates protein degradation through UPS, autophagy, and ERAD	Defects in UBQLN1 lead to perturbed protein degradation via UPS, UBQLN1 downregulation affects APP processing in AD	Cancer, reduced UBQLN1 level found in AD and other neurodegenerative diseases, PolyQ diseases (HD)	Q9UMX0	[6,67]
6	Ubiquilin-2 (UBQLN2)	624	Regulates protein degradation via UPS, autophagy, and ERAD	Defects in UBQLN2 lead to perturbed protein degradation, which leads to neurodegenerative diseases	Mutation in UBQLN2 leads to familial amyotrophic lateral sclerosis (ALS)	Q9UHD9	[26,68,69]
7	Ubiquitin carboxyl-terminal hydrolase isozyme L1 (UCHL1)	223	Processing of ubiquitin precursors and ubiquitinated protein. Maintains pool of mono-Ub	Mutation, dysfunction, and downregulation of UCHL1 affects normal UPS function	Cancer and neurodegenerative diseases such as AD and PD	P09936	[11,31,70]
8	Ubiquitin carboxyl-terminal hydrolase isozyme L5 (UCHL5)	329	Proteasome-associated DUB that cleaves ‘Lys-48’-linked polyubiquitin chains	Upregulation or downregulation of UCHL5	Oncogenesis	Q9Y5K5	[71]
9	Ubiquitin carboxyl-terminal hydrolase 7 (USP7)	1102	Cleaves Ub from polyubiquitin chains of target protein substrate	Poly-Q repeats, mutation, variation in expression level, and dysfunction in USP7	Dysfunction leads to cancer, metabolic and neurological pathologies	Q93009	[10]
10	Ubiquitin carboxyl-terminal hydrolase 14 (USP14)	494	Proteasome-associated DUB that cleaves Ub from Poly-Ub protein before degradation by the proteasome. Negatively regulates proteasome activity.	USP14 activation inhibits degradation of pathogenic, neurotoxic proteins	Neurodegenerative diseases such as AD, ALS, PD, and HD.	P54578	[36,72]
11	Ataxin-3 (ATXN3)	361	DUB that is involved in polyubiquitin chain trimming	PolyQ expansion in ataxin-3 at its C-terminus	Spinocerebellar Ataxia Type 3 (SCA3)	P54252	[73,74]
12	Proteasomal ubiquitin receptor (ADRM1)	407	Receptor for Ub in RP of 26S proteasome that captures target protein by binding to Ub. It also binds and activates DUB enzyme UCHL5	-	-	Q16186	[75]
13	26S proteasome non-ATPase regulatory subunit 2 (PSMD2)	908	Receptor for Ub in RP of 26S proteasome.	-	-	Q13200	[41]
14	26S proteasome non-ATPase regulatory subunit 4 (PSMD4)	377	Receptor for Ub in RP of 26S proteasome, captures target protein by binding to Ub.	-	-	P55036	[41]
15	26S proteasome non-ATPase regulatory subunit 14 (PSMD14)	310	PSMD14 is a 19S-proteasome-associated DUB enzyme that deubiquitinates substrate protein during proteasomal degradation.	Upregulation of PSMD14 leads to dysfunction of UPS	Increase in level of PSMD14 leads to carcinogenesis	O00487	[38,76]

**Table 2:** Predicted percentage of intrinsic disorder (PPID) in the proteins of UPS.

Protein	PPID_VSL2	PPID_VL3	PPID_VLXT	PPID_FIT	PPID_IUPRED	PPID_MEAN
**UBA1 (E1)**	17.86	9.45	22.59	6.14	8.13	9.74
**UBE2R2 (E2)**	48.74	42.44	42.44	28.15	24.79	37.39
**STUB1 (E3)**	41.91	37.62	46.86	34.32	19.80	37.95
**UBB**	30.57	17.03	36.24	7.86	11.35	10.04
**UBQLN1**	88.96	82.17	62.99	85.06	77.76	87.10
**UBQLN2**	86.86	73.40	70.99	81.41	70.83	80.93
**UCHL1**	30.94	11.66	34.98	10.31	3.14	7.62
**UCHL5**	28.27	22.49	25.53	17.02	10.33	16.11
**USP7**	23.96	10.89	19.06	11.62	11.25	11.62
**USP14**	37.25	31.17	26.32	20.85	8.91	23.48
**ATXN3**	58.17	63.16	55.12	47.65	42.38	53.74
**ADRM1**	70.52	60.93	62.16	52.83	51.35	61.92
**PSMD2**	20.37	15.53	27.97	15.86	12.22	13.77
**PSMD4**	63.93	59.15	64.46	47.75	47.21	55.17
**PSMD14**	29.68	20.97	22.26	16.45	13.23	18.71

Proteins and their mean PPIDs are colored to reflect their disorder status (ordered—green, moderately disordered—blue, and highly disordered—red).

**Table 3:** Identification of MoRF regions for the proteins of UPS.

Protein	MoRFCHiBi_Web	MoRFpred	DISOPRED3	ANCHOR
**UBA1 (E1)**	1–13, 1048–1057	5–12, 54–60, 423–427, 1051–1058	802–817	1–16, 23–39
**UBE2R2 (E2)**	166–170, 203–217, 219–226	11–19, 205–213	1–6, 212–238	199–238
**STUB1 (E3)**	-	-	-	163–169, 198–204, 206–214, 230–239
**UBB**	40–50, 116–122, 192–202	221–228	-	-
**UBQLN1**	18–39	34–45, 318–327	1–5, 14–21, 456–474	1–44, 49–54, 72–88, 91–113, 142–168, 192–307, 311–350, 355–496, 507–543
**UBQLN2**	10–38	30–41, 560–565, 588–592	1–19	1–38, 87–107, 136–161, 193–208, 218–329, 353–377, 398–456, 498–598
**UCHL1**	-	215–220	-	-
**UCHL5**	324–328	168–173	1–6, 252–256, 320–329	-
**USP7**	2–26, 1077–1082, 1090–1102	262–267, 511–516, 1094–1099	1084–1093, 1056–1061, 495–505	1–64
**USP14**	-	477–482	66–75, 226–232, 489–494	-
**ATXN3**	56–65, 246–255, 285–290, 312–357	250–254, 282–292, 342–350	1–21, 353–361	215–291, 307–355
**ADRM1**	21–30	24–28, 399–407	1–19, 385–407	140–202, 208–318, 347–383, 399–407
**PSMD2**	1–13, 96–102	51–62, 614–618	-	1–30, 35–79
**PSMD4**	320–345, 365–377	201–205, 329–340, 372–377	196–203, 359–377	201–226, 237–365
**PSMD14**	1–7	1–9, 249–255	1–12, 16–24	-
